# Involvement of LaeA and Velvet Proteins in Regulating the Production of Mycotoxins and Other Fungal Secondary Metabolites

**DOI:** 10.3390/jof10080561

**Published:** 2024-08-08

**Authors:** Xuwen Hou, Liyao Liu, Dan Xu, Daowan Lai, Ligang Zhou

**Affiliations:** MOA Key Lab of Pest Monitoring and Green Management, Department of Plant Pathology, College of Plant Protection, China Agricultural University, Beijing 100193, China; xwhou@cau.edu.cn (X.H.); lyliu@cau.edu.cn (L.L.); cauxudan@cau.edu.cn (D.X.); dwlai@cau.edu.cn (D.L.)

**Keywords:** global regulation, toxigenic fungi, LaeA, velvet proteins, secondary metabolites, mycotoxins, phytotoxins, biosynthetic gene cluster, biological activities, regulation mechanisms

## Abstract

Fungi are rich sources of secondary metabolites of agrochemical, pharmaceutical, and food importance, such as mycotoxins, antibiotics, and antitumor agents. Secondary metabolites play vital roles in fungal pathogenesis, growth and development, oxidative status modulation, and adaptation/resistance to various environmental stresses. LaeA contains an *S*-adenosylmethionine binding site and displays methyltransferase activity. The members of velvet proteins include VeA, VelB, VelC, VelD and VosA for each member with a velvet domain. LaeA and velvet proteins can form multimeric complexes such as VosA-VelB and VelB-VeA-LaeA. They belong to global regulators and are mainly impacted by light. One of their most important functions is to regulate gene expressions that are responsible for secondary metabolite biosynthesis. The aim of this mini-review is to represent the newest cognition of the biosynthetic regulation of mycotoxins and other fungal secondary metabolites by LaeA and velvet proteins. In most cases, LaeA and velvet proteins positively regulate production of fungal secondary metabolites. The regulated fungal species mainly belong to the toxigenic fungi from the genera of *Alternaria*, *Aspergillus*, *Botrytis*, *Fusarium*, *Magnaporthe*, *Monascus*, and *Penicillium* for the production of mycotoxins. We can control secondary metabolite production to inhibit the production of harmful mycotoxins while promoting the production of useful metabolites by global regulation of LaeA and velvet proteins in fungi. Furthermore, the regulation by LaeA and velvet proteins should be a practical strategy in activating silent biosynthetic gene clusters (BGCs) in fungi to obtain previously undiscovered metabolites.

## 1. Introduction

Fungal secondary metabolites are highly complex and have a rich diversity that makes fungi a treasure of bioactive secondary metabolites [1,2,3,4]. Some fungal metabolites are harmful to plants, humans, and animals; these metabolites are usually called mycotoxins [5,6,7,8]. Many bioactive metabolites derived from fungi display their broad potential as agrochemicals, pharmaceuticals, cosmetics, and food additives [9,10].

In recent years, it has become easier, through genome mining, to identify and functionally predict fungal metabolites [11]. Many strategies can regulate (i.e., promote or inhibit) the biosynthesis of secondary metabolites in fungi, such as one strain-many compounds (OSMAC), global regulation, epigenetic regulation, heterologous expression, and combinatorial biosynthesis [12,13,14]. Among them, global regulation for secondary metabolite production has been thought to be the most practical strategy. Global regulation is a complex upper-level regulatory network in which microorganisms respond comprehensively to external environmental stimuli such as light intensity, temperature, ambient pH, redox status, and carbon and nitrogen sources. LaeA and velvet proteins belong to global regulators, and are mainly regulated by light [15], which focus on the secondary metabolism of several fungal species. The velvet proteins, which included VeA, VelB, VelC, VelD and VosA, are widely distributed in the fungal kingdom. At least 21 major velvet clades, with their highly conserved domains, were found in fungi to show functional and type diversity in their velvet proteins [16]. Both LaeA and velvet proteins can form multimeric complexes. They are involved in fungal development and secondary metabolism [15,16,17,18,19,20].

In the past 20 years, many advances have been achieved in regard to the regulation of LaeA and velvet proteins in fungal secondary metabolism and development. Though some reviews have been published [15,16,21], many other recent achievements have not been included. In this review, we focused on the regulation of LaeA and velvet proteins in secondary metabolite production in fungi by either inhibiting the production of mycotoxins, promoting the production of useful metabolites, or revealing previously undiscovered metabolites in order to accelerate their applications.

## 2. Regulation of LaeA on Secondary Metabolite Production in Fungi

LaeA (loss of *aflR* expression) is also called Lae1 or LAE1. It was first identified as a nuclear protein in *Aspergillus*. Compared to the velvet proteins, LaeA has no velvet domain [17]. LaeA functions as a global regulator of secondary metabolism and morphogenetic development in various filamentous fungi. The LaeA protein sequence contains an *S*-adenosylmethionine binding site, so it has been proposed to have methyltransferase activity and might be linked to the remolding of chromatin structure to regulate gene transcription by lysine or arginine methylation of histone [22,23]. In most cases, the *laeA* genes in fungi positively regulated production of secondary metabolites. Only a few *laeA* genes were found to negatively regulate secondary metabolite production (Table 1) [21]. The examples of LaeA regulating secondary metabolite production in fungi are shown in Table 1. The structures of the metabolites are shown in Figure S1.

Overexpression of *AaLaeA* increased production of antitumor compounds, including myricetin (**1**), geraniol (**2**), ergosterol (**3**) and other compounds in the endophytic fungus, *Alternaria alstroemeria* by metabolomic analysis [24]. In contrast, overexpression of *AaLaeA* in another endophytic fungus *A. alstroemeria* derived from the medicinal plant *Artemisia annua* decreased production of antitumor compounds. Unfortunately, these antitumor compounds were not further identified [25]. Many species in the *Alternaria* genus usually belong to the plant pathogenic fungi to produce host-specific toxins (HSTs) and cause host plant diseases [2]. *A. alternata* was the pathogen of some plants, such as tomato, apple and strawberry, helping to produce AAL-, AM-, and AF-toxins, respectively. It was found that LaeA positively regulated production of the HSTs as well as the development and pathogenicity of *A. alternata* [111]. In other instances, deletion of *laeA* in *A. alternata* greatly decreased the production of alternariol (AOH, **4**) and alternariol monomethyl ether (AME, **5**) [26]. Furthermore, overexpression of an LaeA-like global transcriptional regulator in the marine-derived fungus *A. alternata* JJY–32 led to the discovery of an anti-inflammatory meroterpenoid, namely tricycloalternarene O (**6**) [27].

*Arthrobotrys flagrans* (synonym *Duddingtonia flagrans*) was a typical nematode trapping fungus which has been used for nematode biocontrol. Overexpression or deletion of *AfLaeA* positively regulated production of secondary metabolites and their antinematodal activity, whereas some metabolites were not produced due to the absence of *AfLaeA*. The antinematodal activity of these secondary metabolites needed further confirmation [28].

There are many examples of *Aspergillus* species regulated by LaeA to produce secondary metabolities (Table 1). Overexpression of *Az5LaeA* in *Aspergillus* sp. Z5 exhibited increased production of diorcinol (**7**). The *laeA* (*AnLaeA*) gene from *A. nidulans* was heteroexpressed in *Aspergillus* sp. Z5 and was also found to increase production of diorcinol (**7**) [29]. Introduction of *laeA* from *A. nidulans* to *Aspergillus* sp. FKI-5362 led to an increase in production of an antifungal compound MS-347a (**8**) which could inhibit the growth of broad plant pathogenic fungi, including *Botrytis cinerea*, *Colletotrichum gloeosporoides*, *Leptosphaeria maculans*, and *Pyricularia oryzae* [30]. MS-347a (**8**) was previously screened to inhibit myosin light chain kinase from *Aspergillus* sp. KY52178 [112]. When the *laeA* gene was deleted in *A. carbonarius*, the production of citric acid (**9**) [31] and ochratoxin A (OTA, **10**) [32,33] was greatly decreased, respectively. If the deleted mutant Δ*laeA* of *A. carbonarius* was colonized in nectarines and grapes, OTA (**10**) was significantly reduced [33]. When *A. carbonarius* was treated with eugenol at 0.2 μL/mL, OTA (**10**) production was decreased at 87.7%. The transcription of the clustered genes for OTA biosynthesis was significantly reduced under eugenol stress and was further confirmed by RT-qPCR analysis. The expression of LaeA was markedly downregulated by eugenol, which indicated that eugenol was probable through inhibiting LaeA expression to decrease OTA (**10**) production in *A. carbonarius* [33].

Overexpression of *llm1* encoding LaeA-like methyltransferase in *A. cristatus* led to an increase in the contents of multiple secondary metabolites, including terpenoids and flavonoids. Unfortunately, these metabolites were not identified [35]. With a scaled-up fermentation of the Δ*flLaeA* strain of *A. flavipes*, five metabolites, including two previously undescribed piperazine derivatives flavipamides A (**11**) and B (**12**), along with three known nonribosomal peptides asperphenamate (*N*-benzoylphenylalaniny-*N*-benzoylphenyl-alaninate, **13**), 4′-OMe-asperphenamate (**14**), and cyclic Pro-Gly-Val-Gly-Try(8-OH, 3-prenyl)-Gly-Trp (**15**), were obtained by LC-MS guided isolation [36].

Aflatoxins (AFs) have been thought of as the most potent carcinogens, and some fungal species from *Aspergillus* genus are their main producers, especially *A. flavus*. LaeA was revealed to positively regulate production of alfatoxins in *A. flavus* [38,39,40,42]. Deletion of *laeA* in *A. flavus* resulted in the significant upregulation of the NAD^+^-dependent histone deacetylase sirA involved in silencing secondary metabolism clusters via chromatin remodeling. Accompanying the chromatin modification, the enzymes participating in secondary metabolism, including biosynthesis of sterigmatocystin (ST, **16**), aflatoxin B1 (**17**), cyclopiazonic acid (**18**), and ustiloxin B (**19**), were drastically decreased [37,39,41]. The enzymes in the ustiloxin B (**19**) biosynthesis pathway might be indirectedly regulated by LaeA [41]. An interaction partner of LaeA, namely kinetochore protein Spc105, was revealed to regulate development and secondary metabolism in *A. flavus*. Moreover, Spc105 positively regulated the production of secondary metabolites, such as aflatoxins and kojic acid (**20**), and negatively regulated the production of cyclopiazonic acid (**18**). Transcriptome analysis of the Δ*spc105* mutant revealed that 23 backbone genes for secondary metabolism were differentially expressed, corresponding to 19 of the predicted 56 secondary metabolite BGCs, suggesting broad regulatory roles for Spc105 in secondary metabolism in *A. flavus* [43].

The production of several mycotoxins was positively regulated by LaeA in *A. fumigatus*. For examples, the deletion of *laeA* led to decreased production of gliotoxin (**21**), endocrocin (**22**), fumagillin (**23**), fumagatin (**24**), and helvolic acid (**25**) in several strains of *A. fumigatus* [44,45,46,47]. Production of cyclopiazonic acid (**18**) was increased when *laeA* was overexpressed in *A. fumisynnematus* [48]. Deletion of *laeA* gene in *A. luchuensis* mut*. kawachii* led to the reduced production of citric acid (**9**). LaeA was considered as the methyltransferase through regulating the citrate exporter-encoding *cexA* gene [49].

Overexpression of *laeA* in *A. nidulans* led to an increased production of sterigmatocystin (ST, **16**) [54] and terrequinone A (**26**) [50]. Accordingly, the deletion of the *laeA* gene in *A. nidulans* reduced the production of ST (**16**) and norsolorinic aid (NOR, **27**) [51]. The deletion of *laeA* also led to a depressed expression of genes involved in the biosynthesis of ST (**16**), terrequinone A (**26**), and penicillin G (benzylpenicillin, **28**) [52]. However, a contradictory example was that the deletion of *laeA* gene in *A. nidulans* also led to an increased production of ST (**16**) [53]. Generally, LaeA or LaeA-like methyltransferase F (LlmF) negatively regulated biosynthesis of ST (**16**) in *A. nidulans*.

*Aspergillus niger* is a biotechnologically important filamentous fungus and has been thought of as an industrial cell factory for the production of secondary metabolites with a broad spectrum of application fields, covering the agriculture, food, and pharmaceutical industries [113]. Deletion of *laeA* in *A. niger* decreased production of asperrubrol (**29**), atromentin (**30**), and JBIR 86 (**31**) but increased production of aspernigrin A (**32**) and BMS-192548 (**33**), which meant that LaeA positively regulated production of asperrubrol (**29**), atromentin (**30**), and JBIR 86 (**31**) and negatively regulated production of aspernigrin A (**32**) and BMS-192548 (**33**) in *A. niger* [55]. Overexpression of *laeA* gene in *A. niger* led to an activation of secondary metabolite BGCs in the mutant. Three compounds, including flaviolin (**34**), orlandin (**35**) and kotanin (**36**), were identified [56]. LaeA could influence the secondary metabolite profile in *A. niger* FGSC A1279 based on the genome sequencing and transcriptome analysis [114]. The production of ochratoxin A (OTA, **10**) in *A. niger* was decreased in the deleted mutant and increased in the overexpressed mutant. Another similar example was that deletion of *laeA* gene in *A. ochraceus* led to the reduced production of OTA (**10**) [58]. This indicated that LaeA positively regulated the gene expression of the OTA (**10**) BGC in *A. niger* and *A. ochraceus*. In contrast, it was found that the upregulation of gene expression of OTA BGC did not necessarily increase OTA (**10**) production in *A. niger* [57].

Kojic acid (**20**) production was inhibited in the *laeA* disruption strain of *A. oryzae*, and restored in the *laeA* complement strain, which meant that LaeA positively regulated the biosynthesis of kojic acid (**20**) in *A. oryzae* [59]. In the expression system of *A. oryzae*, LaeA also showed its positive regulation on the heterologous BGCs. Overexpression of *laeA* resulted in the increased production of monocolin K (MK, **37**) and terrequinone A (TQ, **38**). The successful production of secondary metabolites belonging to different structural groups, namely MK (**37**) as a polyketide, and TQ (**38**) as a hybrid of amino acid and isoprenoid, indicated that the *laeA*-enriched *A. oryzae* was a versatile host for the heterologous expression of the biosynthetic gene clusters such as the BGC of MK (**37**) from *Monascus pilosus* and the BGC of TQ (**38**) from *A. nidulans* [60]. For *A. pachycristatus* and *A. pseudoterreus*, the production of secondary metabolites was also positively regulated by LaeA [61,62]. Production of sterigmatocystin (**16**) and echinocandin B (ECB, **39**) was decreased in the *laeA* of the deleted mutant of *A. pachycristatus* [61]. Overexpression of *laeA* in *A. pseudoterreus* improved itaconic acid (**40**) yield at the expense of biomass by increasing the expression of key biosynthetic pathway enzymes and attenuating the expression of genes involved in phosphate acquisition and scavenging. Increased yield was observed in optimized conditions as well as conditions containing excess nutrients that might be present in inexpensive sugar containing feedstocks, such as excess phosphate or complex nutrient sources [62].

*A. terrreus* is the main industrial producer of lovastatin (**41**), a drug that lowers cholesterol. Lovastatin (**41**) is also used as a precursor for simvastatin production. In *A. terreus*, the overexpression of the *laeA* gene triggered the increase in gene transcription related to penicillin G (**28**) and lovastatin (**41**) biosynthesis [37]. It has been observed that overexpression of the *laeA* gene in *A. terreus* increased the production of lovastatin (**41**) [63,115]. The chemical epigenetic modifiers 1,3-diaminopropane and spermidine also upregulated lovastatin (**41**) production and expression of lovastatin (**41**) biosynthetic genes in *A. terreus* via LaeA regulation [116]. Overexpression of *laeA* in *A. terreus* resulted in the activation of a silent secondary metabolite cluster without corresponding known metabolites. The yields of two antibacterial alkaloids dihydroisoflavipucines 1 (**42**) and 2 (**43**) reached 183 mg/L and 1.55 mg/mL, respectively. Both compounds showed obviously anti-*Vibrio* activities, with the MIC values ranging from 16 to 64 μg/mL against *Vibrio anguillarum*, *V. campbellii*, *V. harveyi*, and *V. vulnificus* [64].

The production of beauvericin (**44**) and bassiatin (**45**) was reduced in the *BbLaeA* disruption strain of *Beauveria bassiana* but was increased in the overexpressed strain [65].

The production ability of oxalic acid (OA, **46**) was lost in *laeA* disruption strain of *Botryitis cinerea* [66]. Another example was that the production yield of abscisic acid (ABA, **47**) was decreased 90% in the *laeA* disruption strain of *B. cinerea*. It was considered that BcLAE1 was involved in epigenetic regulation as a methyltransferase, with enhanced H3K9me3 modification and attenuated H3K4me2 modification in the Δ*Bclae1* mutant of *B. cinerea* [67].

Overexpression of *laeA* in *Chaetomium globosum* CBS148.51 upregulated expression of the chaetoglobosin BGC and resulted in the isolation of seven cytochalasans, including chaetoglobosins A (**48**), B (**49**), D (**50**), E (**51**), O (**52**), V (**53**), and Z (**54**). Of them, chaetoglobosin Z (**54**) was a new cytochalasan. These cytochalasans displayed strong cytotoxic activity against the HepG 2 cell line [68]. Similarly, the production of chaetoglobusin A (**48**) in the Δ*CglaeA* mutant of another *C. globosum* strain was inhibited, its *CglaeA-C* strain restored the production of chaetoglobusin A (**48**), and the strain of *CglaeA* overexpression led to an increase in chaetoglobusin A (**48**). This indicated that LaeA positively regulated the production of chaetoglobusin A (**48**) in *C. globosum* [69].

*Cladosporium fulvum* was the non-obligate biotrophic fungal tomato pathogen. Deletion of *laeA* in *C. fulvum* led to the increased production of the mycotoxin cladofulvin (**55**), which meant that LaeA negatively regulated biosynthesis of cladofulvin (**55**) in this fungus [70].

T-toxin (**56**) was a host selective phytotoxin produced by the maize pathogen *Cochliobolus heterostrophus*. Deletion of *Chlae1* decreased production of T-toxin (**56**) in *C. heterostrophus* [71].

Coprinoferrin (**57**) was an acylated tripeptide hydroxamate consisting of tandem aligned *N*^5^-hexanoyl-*N*^5^-hydroxy-L-ornithine with modifications of *N*-acetyl and *C*-carboxamide. Knockout of *laeA* in the mushroom fungus *Coprinopsis cinerea* upregulated the biosynthesis of a novel siderophore, namely coprinoferrin (**57**), which indicated that LaeA negatively regulated the production of coprinoferrin (**57**) [72]. The unique chemical properties made coprinoferrin (**57**) an iron (III) binder (siderophore), which helped iron acquisition from the environment and promoted hyphal growth as well as fruiting body formation in *C. cinerea*. In addition, coprinoferrin (**57**) could be chemically synthesized from the *N*-Boc-L-glutamic acid 5-benzyl ester [117].

*Daldinia eschscholzii* was an endophytic fungus from the guts of mantis (*Tenodora aridifolia*). Replacement of the native promoter of the global regulator LaeA-like gene of *D. eschscholzii* by a strong gpdA promoter led to the generation of two novel cyclopentenone metabolites named dalestones A (**58**) and B (**59**). Both dalestones inhibited the gene expression of TNF-α and IL-6 in LPS-induced RAW264.7 macrophages [73].

Deletion of *D*s*LaeA* resulted in enhanced production of dothistromin (**60**) in the pine needle pathogen *Dothistroma septosporum* and increased expression of the regulatory gene *DsAflR* in the dothistromin (**60**) biosynthetic pathway [74].

*Fusarium fujikuroi* (teleomorph: *Gibberella fujikuroi*) is the pathogen of rice bakanae disease that produces a series of secondary metabolites, such as bikaverin (**61**), fusaric acid (**62**), gibberellins, fusarins, and fusarubins. Among them, fusaric acid (**62**) and fusarins belong to the harmful mycotoxins [118]. LaeA positively regulated production of some metabolites in *F. fujikuroi*. For example,, deletion of *laeA* in *F. fujikuroi* led to decreased production of gibberellins A3 (**63**) and A4 (**64**), fusarin C (**65**), fumonisins B1 (**66**), B2 (**67**), B3 (**68**) and B4 (**69**), deoxynivalenol (**70**), and 15-acetyl deoxynivalenol (**71**) [75]. Similar results were subsequently confirmed. Deletion of the *lae1* gene led to reduced production of fusaric acid (**62**), fusarinolic acid (**72**), and dehydrofusaric acid (**73**) in the *F. fujikuroi* strain [76]. Furthermore, deletion of the *lae1* led to decreased production of gibberellins, fumonisins, and fusarin C (**65**). Overexpression of *lae1* led to increased production of gibberelins in another *F. fujikuroi* strain [77]. However, LaeA also negatively regulated production of some metabolites in *F. fujikuroi*. The production of bikaverin (**61**) was increased in the deletion mutant of *F. fujikuroi* [75]. Another example was that deletion of the *lae1* gene in *F. fujikuroi* upregulated the expression of gibepyrone BGC as well as increased the production of gibepyrones A (**74**), B (**75**), C (**76**), D (**77**), E (**78**), and F (**79**) [78].

LaeA positively regulated mycotoxin production of the following phytopathogenic *Fusarium* species. Deletion of *FglaeA* in *F. graminearum* led to a dramatic reduced production of trichothecenes and zearalenone (**80**). Overexpression of *FglaeA* caused the increased production of trichothecenes and zearalenone (**80**). This indicated that FgLaeA positively regulated production of phytotoxins of *F. graminearum* [79]. For the fungus *F. oxysporum*, deletion of *laeA* caused the decreased production of beauvericin (**44**) and fusaric acid (**62**), which contributed to virulence in plant hosts, such as tomato plants [80]. For the fungus *F. oxysporum* f.sp. *niveum*, the deletion of the *FoLae1* gene led to depressed conidiation and reduced production of fusaric acid (**62**) and bikaverin (**70**). In addition, all of these alterations in the deleted mutants were restored in the corresponding complementation strains. [81]. For the fungus *F. verticillioides*, the deletion of *laeA* reduced the production of fusaric acid (**62**), fusarin C (**65**), bikaverin (**70**), and fumonisins [82].

Ganoderic acids (GAs) are lanosterol-type triterpenoids produced by the *Ganoderma* species that possess multiple bioactivities, including anti-cancer, anti-inflammatory, antioxidant, and anti-HIV activities [119]. When a methyltransferase-like laeA gene was deleted in *G. lingzhi*, the production of ganoderic acids was reduced. RT-qPCR analysis further revealed that the transcription levels of genes involved in the biosynthesis of garnoderic acids were drastically lower in the Δ*laeA* strain. In contrast, constitutive overexpression of *laeA* resulted in an increased concentration of GAs [83].

*Magnaporthe oryzae* causes blast disease, the most serious disease of cultivated rice affecting global rice production. *MolaeA* negatively regulated sporulation and melanin biosynthesis and positively regulated production of penicillin G (or called benzylpenicillin, **28**) [84]. Metabolomic profiling analysis showed that overexpression of *MolaeA* led to the increased biosynthesis of secondary metabolites in *M. oryzae*. Unfortunately, these metabolites have not been identified [85].

Some *Monascus* species can produce edible pigments, with their structures bearing a highly oxygenated pyranoquinone bicyclic core and a quaternary carbon center. However, the mycotoxin citrinin (**81**) produced by some *Monascus* strains restricts application of the pigments [120]. Monacolin K (**37**), a cholesterol-lowering agent, was increased three times when *laeA* was overexpressed in *Monascus pilosus*. In addition, the pigment production was also remarkably increased [86]. The production of monacolin K (**37**) was also increased when *laeA* was overexpressed in *M. purpureus* [87]. For another *Monascus* species, the deletion of *MrlaeA* in *M. ruber* exhibited a drastically reduced production of toxin citrinin (CIT, **81**) and six pigments, namely rubropunctamine (**82**), monascorubramine (**83**), monascin (**84**), rubropunctatin (**85**), ankaflavin (**86**), and monascorubrin (**87**) [88].

The *laeA* gene from *Aspergillus nidulans* was heteroexpressed in the fungus *Penicillium* sp. LC1-4. Overexpression of *AnLaeA* caused an increased production of a bioactive compound quinolactacin A (**88**). It indicated that heteroexpressed of AnLaeA in fungi was a simple and effective method to explore metabolic potential [29]. LaeA could also positively regulate production of antibacterial pseurotins in *Penicillium* sp. Deletion of the *laeA* gene in *Penicillium* sp. strain MB inhibited production of the members with a 1-oxa-7-aza-spiro [4,4] non-2-ene-4,6-dione skeleton. Among these deduced compounds, pseurotins A (**89**), B (**90**), C (**91**), D (**92**), and E (**93**) displayed obvious antibacterial activity. This was why cheese rind bacterial communities assembled with the *laeA* deletion mutant of *Penicillium* sp. strain MB had significantly higher bacterial abundances than the wild-type strain [89].

To date, secondary metabolite production in various *Penicillium* species has been found to be positively regulated by LaeA. Overexpression of *PbrLaeA* led to the discovery of four compounds, namely fumigatin chlorohydrin (**94**), iso-fumitatin chlorohydrin (**95**), spinulosin (**96**), and pyranonigrin F (**97**), in the fungus *P. brocae* HDN-12-143. Among them, iso-fumitatin chlorohydrin (**95**) was a new compound. Both fumigatin chlorohydrin (**94**) and iso-fumitatin chlorohydrin (**95**) exhibited cytotoxic activity against HL-60, with IC_50_ values of 18.63 µM and 24.83 µM, respectively [90]. Overexpression of *PclaeA* in *P. chrysogenum* gave rise to a 25% increase production of penicillin G (benzylpenicillin, **28**). *PclaeA* knock-down mutants exhibited drastically reduced production and biosynthesis gene expression of penicillin G (**28**) [22]. Deletion of *laeA* in *P. chrysogenum* decreased production of penicillin G (**28**) [91]. However, epigenetic modifiers 1,3-diaminopropane (1,3-DAP) and spermidine completely restored the levels of penicillin G production in the *laeA* knock-down mutant. This indicated that LaeA in *P. chrysogenum* might act epigenetically on the expression of secondary metabolite genes by heterochromatin reorganization, which should be studied in detail [92]. Small reduction in penicillin G (**28**) was also reported in another Δ*PclaeA* mutant of *P. chrysogenum* [93]. The full-length *laeA* gene, namely *Pci-laeA*, with the sequence as 1340 bp, including an ORF of 1284 bp encoding 427 amino acids, was cloned from *P. citrinum*. The predicted molecular mass of Pci-LaeA was 48.72 kDa, with an estimated theoretical isoelectric point of 6.96. Pci-LaeA had a conserved *S*-adenosylmethionine binding site and a potential MlcR (a pathway specific regulator in mevastatin biosynthesis) binding site [121]. When an *laeA* gene was deleted in *P. citrinum*, production of compactin (also named ML-236B, mevastatin, **98**) was suppressed [94]. Comparative transcriptome analysis revealed that the function loss of *PdLaeA* in *P. digitatum* resulted in the reduced expression of several secondary metabolite gene clusters [95].

Sorbicillinoids are important hexaketide metabolites derived from fungi. They have a variety of biological activities with unique structural features to make them attractive candidates for developing new pharmaceutical and agrochemical agents [122,123]. Overexpression of the *laeA* gene in the marine-derived fungus *P. dipodomyis* YJ-11 induced metabolic variations to afford a series of sorbicillinoids, including two new ones named 10,11-dihydrobislongiquinolide (**99**) and 10,11,16,17-tetrahydrobislongiquinolide (**100**), as well as four known analogues, bislongiquinolide (**101**), 16,17-dihydrobislongiquinolide (**102**), sohirnone A (**103**), and 2′,3′-dihydrosorbicillin (**104**). This indicated that regulation of LaeA is a useful strategy in activating silent gene clusters in fungal strains to obtain previously undiscovered compounds [96].

The mycotoxin patulin (**105**) was produced in the colonized tissue by *P. expansum* during the storage of apples. Deletion of *laeA* in *P. expansum* led to a decrease in patulin (**105**) production, which positively regulated patulin gene expression and patulin biosynthesis. Loss of LaeA affected the colonization of *P. expansum* in apple fruits. The Δ*laeA* strains showed reduced virulence at all stages of apple maturity, and the disease severity was reduced by up to 22% in more mature fruits [97,98]. This demonstrated that patulin metabolism modulated by LaeA contributed in part to the pathogenicity of *P. expansum* [97].

The LaeA in *P. oxalicum* played an important role in asexual development, the expression of secondary metabolite gene clusters, and extracellular glycoside hydrolase synthesis. Deletion of the *laeA* gene led to decreased production of secondary metabolites. Unfortunately, these differential metabolites have not been identified [99]. Four (i.e., cluster_1, cluster_5, cluster_14, and cluster_26) of the 28 secondary metabolic gene clusters were significantly downregulated in the Δ*laeA* mutant compared with the wild-type strain (WT) of *P. oxalicum*. The LaeA was speculated to be the putative methyltransferase. Histone H2B lysine 122 and lysine 130 were considered as the putative targets of LaeA [100]. Another example was that the disruption of *PrlaeA* in *P. roqueforti* led to a substantial reduction in the production of the three metabolites roquefortine C (**106**), mycophenolic acid (**107**), and andrastin A (**108**). However, deletion of *PrlaeA* had little impact on asexual development [101].

Disruption of *laeA* in the *Pestalotiopsis microspore* led to the decreased production of pestalotiollide B (PB, **109**) [102]. Similarly, deletion of *PoLaeA1* in *Pleurotus ostreatus* decreased the intracellular polysaccharide (IPS) content by about 28–30% as well as the cellulose activity, which provided new insights into the regulation of polysaccharide biosynthesis and cellulose production in filamentous fungi [103]. *PoLAE1* also positively regulated tenuazonic acid (TeA, **110**) production of the rice blast pathogen *Pyricularia oryzae* (teleomorph: *Magnaporthe oryzae*) [104].

LaeA positively regulated the secondary metabolite production of the following *Trichoderma* species. Overexpression of *TalaeA* in *T. afroharzianum* led to the production of two new antifungal polyketides: (1*R*,3*E*,5*E*)-1-(3,5-dihydroxy-2,4-dimethylphenyl)-1-hydroxyhepta-3,5-dien-2-one (**111**) and (1*R*,3*E*,5*E*)-1-(3,5-dihydroxy-2,4-dimethylphenyl)-1-methoxyhepta- 3,5-dien-2-one (**112**). Both compounds showed strong antifungal activity on plant pathogenic fungi *Botrytis cinerea*, *Colletotrichum lagenarium*, and *Fusarium oxysporum* f.sp. *nicotianae* [105]. Deletion of *Tllae1* in *T. longibrachiatum* reduced the production of peptaibols to a large degree. The peptaibols belonged to antimicrobial peptides and were named as trichokonins (TKs) which were mainly classified into 20-aa trichokonin A (TKA) and 12-aa trichokonin B (TKB). Overexpression of *Tllae1* in *T. longibrachiatum* led to two-fold increased production of petaibols. Overexpression of *laeA* gene in *T. reesei* led to the increased production of sorbicillinoids, which were not identified. If the *laeA* gene in another *T. reesei* strain was deleted, the production of sterigmatocystin (**16**) was decreased [108]. *T. reesei* had a potential to produce terpenoids. If the *lae1* gene along with major hemi-cellualse genes were deleted, the production of sesquiterpenoid ophiobolin F (**113**) in *T. reesei* was increased to 1187.06 mg/L by using the modified chassis [109].

Deletion of *VmLaeA* in apple canker pathogen *Valsa mali* led to greatly reduced virulence, with lesion length being reduced by 48% in apple twigs. The toxicity of secondary metabolites produced by *VmLaeA* deletion mutant (Δ*VmlaeA*) was markedly decreased in comparison with the wild-type strain. Unfortunately, these toxic metabolites have not been identified [110].

## 3. Regulation of Velvet Proteins on Secondary Metabolite Production in Fungi

The velvet proteins (or so-called velvet family proteins) included VeA (velvet A), VelB (velvet like B), VelC (velvet like C), VelD (velvet like D), and VosA (viability of spores A). These five proteins all contain the velvet and transactivation domains. They are highly conserved in dimorphic and filamentous fungi [16,39,124,125,126]. They mainly play important roles in fungal development, asexual sporulation, sexual development, secondary metabolism, and stress tolerance [127]. It has been revealed that LaeA and velvet proteins formed multimeric complexes, such as VelB-VeA-LaeA, VelB-VosA, and VelB-VelB, in fungi. The heterotrimeric VelB-VeA-LaeA complex controls sexual development and secondary metabolism in response to light [128,129,130,131,132]. The following is the research progress of velvet proteins on the regulation of secondary metabolite production in fungi.

### 3.1. Regulation of VeA on Secondary Metabolite Production in Fungi

The VeA (also called VelA, Ve1, and Vel1) proteins usually positively regulated production of secondary metabolites in fungi. Most of the regulated metabolites were polyketides. Some examples of VeA proteins regulating secondary metabolite production in fungi are shown in Table 2. The structures of the metabolites are shown in Figure S1.

Disruption of the *AcveA* gene in *Acremonium chrysogenum* resulted in a reduction in cephalosporin C (**114**) production, which meant that AcVeA positively regulated cephalosporin C (**114**) biosynthesis in *A. chrysogenum* [133].

Deletion of the *veA* gene in *Alternaria alternata* greatly reduced sporulation and the production of alternariol (AOH, **4**) and alternariol monomethyl ether (AME, **5**) [26]. The production of both AOH (**4**) and AME (**5**) in *A. alternata* was significantly stimulated by blue light. The disruption of *AaVeA* resulted in a marked decrease in AOH (**4**) and AME (**5**) production under blue light illumination [134].

*Aspergillus carbonarius* was the pathogen of grape Aspergillus rot [162]. The fungus could produce ochratoxin A (OTA, **10**). Upon the deletion of *veA* in *A. carbonarius*, the production of OTA (**10**) almost disappeared [32]. Deletion of *veA* in *A. carbonarius* resulted in significant reduction in OTA (**10**) production. During both in vitro growth and the infection of grapes, non-mycotoxigenic strains could outcompete the wild-type strain. The OTA (**10**)-defective Δ*veA* mutant was considered as the potential biocontrol agent [135].

VeA affected the biosynthesis of mycotoxins in *Aspergillus flavus*. Deletion of *veA* in *A. flavus* decreased the production of cyclopiazonic acid (**18**), aflatrem B (**115**), and aflatoxins [136]. Deletion of *veA* also decreased the production of asparasone A (**116**) [137] and aflatoxin B1 (**17**) in *A. flavus* [138]. VepN contained a septin-type guanine nucleotide-binding domain, representing a conserved protein family from yeast to humans belonging to the P-loop GTPase superfamily. It was found that the global regulation gene *veA* positively regulated *vepN* to influence aflatoxin production, morphological development, and pathogenicity in *A. flavus* [139].

Both deletion and overexpression of *veA* in *Aspergillus fumigatus* led to decreased production of gliotoxin (**21**). The RNA sequencing data provided evidence supporting this pattern. It was possible that both deletion and overexpression of *veA* downregulated *fumR* transcription, suggesting that *veA* influenced the activation of the fumagillin gene cluster through regulation of *fumR* [140]. The similar regulation pattern was also observed in *A. fumigatus* to produce other secondary metabolites. Both deletion and overexpression of *veA* in *A. fumigatus* decreased the production of fumagillin (**23**), gumitremorgin G (**117**), fumigaclavine C (**118**), and glionitrin A (**119**) [141].

The deletion of *veA* in *A. nidulans* suppressed the production of sterigmatocystin (**16**). The *veA* deletion mutant produced less penicillin G (**28**) than the regular strain. The *veA* gene was also required for sexual development [142]. The deletion of the *veA* gene in *A. nidulans* reduced the production of sterigmatocystin (**16**) and norsolorinic aid (NOR, **27**) [51]. VeA was thought to be involved in the penicillin G (**28**) biosynthesis via repression of the expression of the *acvA* gene, which led to reduced penicillin production in *A. nidulans* [143]. Upon further investigation, it was found that VeA repressed the expression of the cryptic orsellinic acid (**120**) BGC in *A. nidulans* through histone 3 acetylation. The deletion of *veA* led to increased production of orsellinic acid (**120**), F9775A (**121**), and F9775B (**122**) in *A. nidulans* [144]. Further investigation revealed that *A. nidulans* VeA was a multi-phosphorylated protein, and it was hypothesized that at least four specific amino acids (T167, T170, S183 and Y254) underwent reversible phosphorylation to trigger development and sterigmatocystin (**16**) biosynthesis. Double mutation of T167 to valine and T170 to glutamic acid exerted the largest effects with regard to sexual development and *veA* gene expression [163]. In the dark, VeA entered the nucleus of *A. nidulans*, formed a VelB-VeA-LaeA heterotrimeric complex, controlled sexual development, and enhanced sterigmatocystin (**16**) production [128]. The downstream transcription factor regulatory gene of *veA* was revealed as *mtfA* in *A.nidulans*. Deletion of *mtfA* could decrease the expression of the genes in the penicillin gene cluster, reducing penicillin production. In this case, overexpression of *mtfA* enhanced the transcription of the penicillin BGC, increasing penicillin production more than five-fold with respect to the control. However, it was detrimental for the expression of the terrequinone BGC in regard to either deletion or overexpression of mtfA. In addition to its effect on secondary metabolism, mtfA also affected asexual and sexual development in *A. nidulans*. Deletion of *mtfA* resulted in a reduction in the conidiation and sexual stages [164]. Another example of the downstream transcription factor regulatory gene of *veA1* was revealed in *sclB* in *A. nidulans*. Deletion of *sclB* also decreased production of aspernidines [145].

The deletion of *veA* in *Aspergillus niger* suppressed the production of ochratoxins A (OTA, **10**), α (OTα, **123**), and β (OTβ, **124**). The *veA* gene acted as the positive regulator of conidia production, OTA (**10**) biosynthesis, and oxidative stress tolerance in *A. niger* regardless of light conditions. Darkness promoted conidial production and OTA (**10**) biosynthesis in the wild-type strain of *A. niger* [146]. The deletion of *veA* in *A. oryzae* also decreased production of kojic acid (**20**) [147]. Disruption of *veA* significantly reduced the production of echinocandin B (**39**) and sterigmatocystin (**16**) in *A. pachycristatus* [61]. Deletion of the *veA* gene in *A. parasiticus* reduced aflatoxin BGC gene expression and aflatoxisome development [149].

CgVeA in *Chaetomium globosum* was thought of as a light-signaling responsive regulator. It was involved in the regulation of chaetoglobusin A (**48**) biosynthesis. Deletion of *CgveA* caused an obvious decrease in chaetoglobusin A (**48**) production from 51.32 to 19.76 mg/L under dark conditions. In contrast, *CgveA* overexpression resulted in a dramatic increase in chaetoglobusin A (**48**) production, reaching 206.59 mg/L under illumination, which was higher than that noted in darkness. The RT-qPCR results confirmed that CgVeA, as a light-responsive regulator, positively regulated chaetoglobusin A (**48**) biosynthesis by controlling the expression of core genes of the chaetoglobusin A (**48**) biosynthetic gene cluster and other relevant regulators [150].

Deletion of *veA* in tomato fungal pathogen *Cladosporium fulvum* led to increased production of the pigment cladofulvin (**55**), which meant that VeA negatively regulated biosynthesis of cladofulvin (**55**) in this fungus [70].

Deletion of *veA* in *Fusarium fujikuroi* led to decreased production of gibberellins A3 (**63**) and A4 (**64**), fusarin C (**65**), fumonisins B1 (**66**), B2 (**67**), B3 (**68**) and B4 (**69**), deoxynivalenol (**70**), and 15-acetyl deoxynivalenol (**71**). However, the production of bikaverin (**61**) was increased in the deletion mutant [75]. The similar results were confirmed later. Deletion of *vel1* led to reduced production of fusaric acid (**62**), fusarinolic acid (**72**), and dehydrofusaric acid (**73**) in the *F. fujikuroi* strain [76]. Deletion of *vel1* led to decreased production of gibberellins, fumonisins, and fusarin C (**65**). Overexpression of *lae1* led to increased production of gibberelins in another *F. fujikuroi* strain [77]. Deletion of the *vel1* gene in *F. fujikuroi* led to upregulation of gibepyrone BGC expression as well as the increased production of gibepyrones A (**74**), B (**75**), C (**76**), D (**77**), E (**78**), and F (**79**) [78].

Deletion of *veA* in *Fusarium graminearium*, the causal agent of Fusarium head blight, led to reduced production of deoxynivalenol (vomintoxin or DON, **68**) [151] and decreased production of trichothecenes [152].

Upon the overexpression of *FnveA* in *Fusarium nematophilum*, the antitumor activity of the crude extract was increased on A549 cancer cells. Unfortunately, the antitumor compounds were not identified [153].

Deletion of *veA* in *Fusairum oxysporum* caused the decreased production of beauvericin (**44**) and fusaric acid (**62**), which contributed to virulence on plant hosts such as tomato plants [80]. Deletion of the *FoVel1* gene in *F. oxysporum* f.sp. *niveum* led to depressed conidiation and reduced production of bikaverin (**61**) and fusaric acid (**62**). In addition, all of these alterations in the deleted mutants were restored in the corresponding complementation strains [81].

Deletion of *Ffvel1* in *Fusarium verticillinoides* led to decreased production of gibberellins (**63**) and A4 (**64**), fusarin C (**65**), fumonisins B1 (**66**), B2 (**67**), B3 (**68**) and B4 (**69**), deoxynivalenol (**70**), and 15-acetyl deoxynivalenol (**71**). However, the production of bikaverin (**61**) was increased in the deletion mutant. The regulation mechanisms of *vel1* in the above metabolite production should be similar to those of *laeA* in this fungus [75]. Deletion of *veA* in maize pathogen *Fusarium verticillioides* led to decreased production of fusarin C (**65**) and fumonisins B1 (**66**), B2 (**67**), and B3 (**68**) [154]. Further investigation showed that VeA was necessary for causing symptom and mycotoxin synthesis in maize seedlings by *F. verticillioides* [165].

Deletion of the *mve1* gene in *Mycosphaerella graminicola* decreased production of melanin. The Δ*mve1* mutant displayed an albino phenotype with a significant reduction in melanin biosynthesis and less production of aerial mycelia on agar plates [155].

Deletion of the *veA* gene in *Neurosopora crassa* decreased both asexual conidiation and carotenoid production [156]. Further investigation showed that the production of siderophore coprogen (**125**) was also decreased in the Δ*veA* mutant of *N. crassa* [157].

Deletion of the *veA* gene in *Penicillium chrysogenum* decreased the production of penicillin G (**28**) [91,158]. A small reduction in penicillin G (**28**) was also reported in another Δ*PcvelA* mutant of *P. chrysogenum* [93]. *P. citrinum* is known to produce compactin (also called ML-236B or mevastatin, **98**). This polyketide exhibited a potent inhibitory activity on 3-hydroxy-3-methylglutaryl-coenzyme A (HMG-CoA) reductase. Compactin (**98**) was industrially converted into pravastatin by microbes. Currently, pravastatin has been widely used as a pharmaceutical drug for the treatment of hypercholesterolemia [158]. Deletion of *veA* in *P. citrinum* led to suppressed production of compactin (**98**), and overexpression of *veA* led to increased production of compactin (**98**). This indicated that *veA* is the key regulation factor of compactin (**98**) biosynthesis [94]. Overexpression of *veA* in *P. citrinnum* led to compactin (**98**) production being increased [159].

*Penicillium expansum* is the causal agent of apple blue mold disease [166]. It produces the mycotoxins citrinin (**81**) and patulin (**105**). The disruption of *veA* in *P. expansum* drastically reduced the production of citrinin (**81**) and patulin (**105**) in synthetic media, which is associated with a marked downregulation of all genes involved in the biosynthesis of the two mycotoxins. Moreover, the null mutant Δ*PeveA* strain was unable to produce patulin (**105**) in apples [127]. Deletion of *veA* in *P. expansum* led to the production of patulin (**105**) in the Δ*veA* mutant being completely blocked [160,161]. The Δ*veA* mutants also exhibited reduced growth and conidiation when exposed to stressors, including cell membrane stress, oxidative stress, osmotic stress, and different pH values, which indicated that patulin (**105**) contributed to the fungal anti-stress ability. The non-mycotoxigenic strain Δ*veA* of *P. expansum* showed its biocontrol capability against a postharvest pathogen of pome fruit during postharvest handling and storage [161].

Deletion of the *veA* gene in *Pestalotiopsis microspora* led to increased production of pestalotiollide B (**103**). The *veA* gene appeared to negatively regulate the biosynthesis of pestalotiollide B (**103**) [102].

### 3.2. Regulation of VelB on Secondary Metabolite Production in Fungi

VelB (also called Vel2) mainly coordinates with other members, such as LaeA, VeA and VosA, to regulate the production of fungal secondary metabolites in fungi, as VelB lacks a site for a nuclear localization signal (NLS) [128]. Some examples of VelB regulating secondary metabolite production in fungi are shown in Table 3. The structures of the metabolites are shown in Figure S1.

Deletion of *velB* in *Aspergillus flavus* abolished aflatoxin production and sclerotial formation either under illumination or in darkness. VelB may have a dual role and likely coordinates with FluG to modulate its functions [167]. A similar result was reported by Eom et al., indicating that inactivation of *velB* led to decreased production of aflatoxin B1 (**17**) in *A. flavus* [138].

The knock out of the *velB* gene in *Aspergillus nidulans* led to reduced content in sterigmatocystin (**16**) under illumination. However, the production yield of sterigmatocystin (**16**) in the deletion mutant was almost the same as that of the wild-type strain, which indicated that the mycelial growth rate of the deletion mutant was bigger than that of the wild-type strain [128]. Further investigation showed that the deletion of *velB* in *A. nidulans* resulted in decreased mRNA levels of *vadJ* throughout the life cycle. Conversely, the deletion of *vadJ* resulted in elevated production of sexual fruiting bodies and sterigmatocystin (**16**). This indicated that *velB* was necessary for proper coordination via *vadJ* to regulate sterigmatocystin (**16**) production [168].

Deletion of the *velB* gene in *Aspergillus ochraceus* led to drastically reduced production of ochratoxin A (OTA, **10**) [58].

The deletion of *BcvelB* led to increased conidiation and melanin biosynthesis in *Botrytis cinerea*. The expression of the melanin biosynthesis gene cluster was also upregulated [169].

CsVelB positively regulated the melanin production of *Colletotrichum siamense* [170].

Deletion of the *ClvelB* gene in *Curvularia lunata* led to a decrease in the production of conidia and the phytotoxin methyl 5-hydroxymethylfuran-2-carboxylate (**126**). The Δ*ClvelB* mutant was impaired in colonizing the host tissue. However, deletion of the *ClvelB* gene led to an increase in aerial hyphae and melanin production [176].

Deletion of the *vel2* gene in *F. fujikuroi* led to upregulation of the gibepyrone BGC expression as well as increased production of gibepyrones A (**74**), B (**75**), C (**76**), D (**77**), E (**78**), and F (**79**) [78].

Deletion of *FgvelB* in *Fusarium graminearum* led to decreased production of deoxynivalenol (DON, **68**) [171]. Production of trichothecenes and zearalenone (**80**) in the *FgvelB*-deleted strain of *F. graminearum* was also dramatically reduced compared with the wild strain [172]. A similar example is that the deletion of *FpvelB* led to notable differences in growth, conidiation, virulence, and deoxynivalenol (**68**) production in *F. pseudograminearum*. Furthermore, FpVelB positively regulated another secondary metabolite BGC associated with pathogenesis by modulating the expression of the *PKS11* gene. FpVelB regulated the pathogen virulence by influencing deoxynivalenol (**68**) production in *F. pseudograminearum* [173].

Deletion of *velB* in *Neurospora crassa* led to reduced biosynthesis of light-dependent carotenoids [157].

*Penicillium expansum* is the pathogen of apple blue mold disease and the main producer of patulin (**105**). The Δ*PevelB* mutant colonized apples, albeit at a lower rate than the wild-type and complemented strains. Conidiation was significantly reduced in the Δ*PevelB* strain. Under light conditions, the Δ*PevelB* strain showed a reduced level of spore viability. Deletion of the *velB* gene strongly inhibited the production of mycotoxins chaetoglobusin A (**48**), citrinin (**81**), and patulin (**105**) in synthetic media or in planta but increased the production of fumarylalanine (**127**). In addition, the genes involved in siderophore biosynthesis, ergosterol biosynthesis, and nitrate assimilation were also upregulated in the Δ*PevelB* strain. This indicated that VelB was involved in the development, pathogenicity, and secondary metabolism of *P. expansum* [175].

Deletion of *veA* in *Pestalotiopsis microspora* led to a decrease in production of pestalotiollide B (**103**). The *velB* gene appeared to stimulate the biosynthesis of pestalotiollide B (**103**) [102].

### 3.3. Regulation of VelC, VelD and VosA on Secondary Metabolite Production in Fungi

In velvet proteins, the functions of VelC, VelD, and VosA in fungi have been seldom studied for their regulation in secondary metabolism. Some examples of VelC, VelD, and VosA regulating secondary metabolite production in fungal species of the genera *Aspergillus* and *Penicillium* are shown in Table 4. The structures of the metabolites are shown in Figure S1.

#### 3.3.1. Regulation of VelC

The VelC (also called Vel3 and VE-3) protein belongs to the velvet family of regulators involved in the control of development and secondary metabolite production in fungi [180]. Deletion of the *velC* gene in *Aspergillus oryzae* led to decreased production of kojic acid (**20**) [147]. Deletion of the *velC* gene in *Penicillium expansum* led to markedly decreased production of patulin (**105**) [160]. For the above-mentioned two fungal species, VelC positively regulated secondary metabolite production.

#### 3.3.2. Regulation of VelD

The VelD protein is also known as Vel4. Deletion of the *velD* gene in *Aspergillus flavus* led to decreased production of aflatoxin B1 (**17**) [138]. Another example was that deletion of the *velD* gene in *A. oryzae* led to decreased production of kojic acid (**20**) [147]. It indicated that VelD positively regulated secondary metabolite production in *A. flavus* and *A. oryzae*.

#### 3.3.3. Regulation of VosA

The regulation of VosA in secondary metabolite production was studied in detail via a sample of *Aspergillus nidulans*. The deletion of *vosA* in *A. nidulans* resulted in a lack of trehalose (**128**) in spores, a rapid loss of the cytoplasm, organelles, and viability of spores, and a dramatic reduction in the tolerance of conidia to heat and oxidative stress [177]. RNA-seq-based genome-wide expression analysis demonstrated that the loss of *vosA* in *A. nidulans* led to elevated expression of sterigmatocystin (**16**) biosynthetic genes and a slight increase in sterigmatocystin (**16**) production in ascospores. Moreover, the deletion of *vosA* caused the upregulation of additional gene clusters associated with the biosynthesis of other secondary metabolites, including asperthecin (**129**), microperfuranone (**130**), and monodictyphenone (**131**) [178]. VosA in *A. nidulans* could interact with the downstream target SclB to negatively regulate production of secondary metabolites, including emericellamides A (**132**), C (**133**), and D (**134**), as well as austinol (**135**) and dehydroaustinol (**136**) [125]. The second instance of such a scenario was that the VosA-repressed *dnjA* gene negatively regulated metabolism in the *Aspergillus* species. The deletion of *dnjA* caused increased production of sterigmatocystin (**16**) and aflatoxin B1 (**17**) in *A. nidulans* and *A. flavus*, respectively [181]. The third instance was that the VosA-VelB-repressed *mcrA* gene negatively regulated sterigmatocystin (**16**) production in *A. nidulans*. The conidia of the Δ*mcrA* mutant contained more amounts of sterigmatocystin (**16**) [182]. The fourth instance involved the VosA-VelB targeted gene *vidD*, which was required for proper fungal growth, development, and sterigmatocystin (**16**) production in *Aspergillus nidulans* [183]. Furthermore, transcriptomic, protein–DNA interaction, and metabolomics studies of VosA, VelB, and WetA in *A. nidulans* played interdependent, overlapping, and distinct roles in governing morphological development and metabolic remodeling in the conida, leading to the production of vital conidia suitable for fungal proliferation and dissemination. The related secondary metabolites regulated by VosA, VelB, and WetA in *A. nidulans* asexual spores included sterigmatocystin (**16**), austinol (**135**), dehydroaustinol (**136**), norsolorinic acid (**137**), nidurufin (**138**), versiconol (**139**), and emericellamids A (**132**), C (**133**), D (**134**), E (**140**), and F (**141**) [184]. In addition, Vos-VelB could activate putative C_6_ transcription factor VadZ to regulate development and sterigmatocystin (**16**) production in *A. nidulans* [179].

## 4. Conclusions

LaeA and velvet proteins could obviously regulate the production of fungal secondary metabolites by responding to the light conditions under which fungi are grown. We can manipulate fungal secondary metabolite production to inhibit the production of harmful mycotoxins while promoting the production of useful metabolites [157]. However, we only know a little about the regulation mechanisms between LaeA/velvet proteins and secondary metabolite BGC expression, which should be studied in detail in the future [11,14,64,185].

In summary, the regulation studies of LaeA mainly focus on the toxin-producing fungal species for control of mycotoxin production, as well as plant endophytic fungi and marine-derived fungi for mining novel bioactive compounds. This should be an effective strategy for promoting or inhibiting production of secondary metabolites through global regulation of LaeA and velvet proteins in fungi. Some cryptic BGCs for secondary metabolite production are possibly activated by LaeA and velvet proteins through the regulatory networks [68,90,96,105]. This is beneficial for the excavation of bioactive compounds from fungi. Furthermore, some non-mycotoxigenic fungal strains obtained by deletion or overexpression of *laeA* or velvet protein encoding genes could be used as the biocontrol agents during the application to plants to reduce mycotoxin contamination [161].

## Figures and Tables

**Table 1:** Some examples of LaeA regulating secondary metabolite production in fungi.

Fungus	Overexpression/ Deletion of *laeA*	Positive/Negative Regulation	Production of Secondary Metabolites	Ref.
*Alternaria* *alstroemeria*	Overexpression	Positive	Increased production of myricetin (**1**), geraniol (**2**), ergosterol (**3**) and other compounds determined by metobolomic analysis.	[24]
*Alternaria* *alstroemeria*	Overexpression	Negative	Decreased production of the antitumor compounds via controlling the transcription of *AaFla1*.	[25]
*Alternaria* *alternata*	Deletion	Positive	Decreased production of alternariol (**4**) and alternariol monomethyl ether (**5**).	[26]
*Alternaria* *alternata*	Overexpression	Positive	Increased production of the anti-inflammatory meroterpenoid tricycloalternarene O (**6**).	[27]
*Arthrobotrys* *flagrans*	Overexpression and deletion	Positive	Increased production of the secondary metabolites by overexpression of *AflaeA*, and decreased production of the secondary metabolites by deletion of *AflaeA*.	[28]
*Aspergillus* sp. Z5	Overexpression	Positive	Increased production of diorcinol (**7**).	[29]
*Aspergillus* sp. FKI-5362	Overexpression	Positive	Increased production of MS-347a (**8**).	[30]
*Aspergillus* *carbonarius*	Deletion	Positive	Decreased production of citric acid (**9**).	[31]
*Aspergillus* *carbonarius*	Deletion	Positive	Decreased production of ochratoxin A (**10**).	[32]
*Aspergillus* *carbonarius*	Deletion	Positive	Decreased production of ochratoxin A (**10**) in Δ*laeA* strain colonized in nectarines and grapes.	[33]
*Aspergillus* *carbonarius*	Inhibtion of LaeA	Positive	Decreased production of ochratoxin A (**10**) by treatment with eugenol through inhibiting LaeA expression.	[34]
*Aspergillus* *cristatus*	Overexpression	Positive	Increased production of multiple secondary metabolites including terpenoids and flavonoids.	[35]
*Aspergillus flavipes*	Deletion	Negative	Increased production of flavipamides A (**11**) and B (**12**), asperphenamate (**13**), 4′-OMe-asperphenamate (**14**), and cyclic Pro-Gly-Val-Gly-Try(8-OH, 3-prenyl)-Gly-Trp (**15**).	[36]
*Aspergillus* *flavus*	Deletion	Positive	Decreased production of sterigmatocystin (**16**).	[37]
*Aspergillus* *flavus*	Deletion	Positive	Decrease production of aflatoxins.	[38]
*Aspergillus* *flavus*	Deletion	Positive	Decreased production of aflatoxin B1 (**17**).	[39]
*Aspergillus* *flavus*	Deletion	Positive	Decreased production of aflatoxins.	[40]
*Aspergillus* *flavus*	Deletion	Positive	Decreased production of aflatoxins, cyclopiazonic acid (**18**) and ustiloxin B (**19**).	[41]
*Aspergillus* *flavus*	Deletion	Positive	Decreased production of aflatoxins.	[42]
*Aspergillus* *flavus*	Deletion	Positive	Decreased production of aflatoxins and kojic acid (**20**).	[43]
*Aspergillus* *fumigatus*	Deletion	Positive	Decreased production of gliotoxin (**21**) and endocrocin (**22**).	[37]
*Aspergillus* *fumigatus*	Deletion	Positive	Decreased production of several mycotoxins including gliotoxin (**21**).	[44]
*Aspergillus* *fumigatus*	Deletion	Positive	Decreased production of gliotoxin (**21**), fumagillin (**23**), fumagatin (**24**) and helvolic acid (**25**).	[45]
*Aspergillus* *fumigatus*	Deletion	Positive	Decreased production of gliotoxin (**21**).	[46]
*Aspergillus* *fumigatus*	Deletion	Positive	Decreased production of endocrocin (**22**).	[47]
*Aspergillus* *fumisynnematus*	Overexpression	Positive	Increased production of cyclopiazonic acid (**18**).	[48]
*Aspergillus* *luchuensis* mut. *kawachii*	Deletion	Positive	Decreased production of citric acid (**9**).	[49]
*Aspergillus* *nidulans*	Overexpression	Positive	Increased production of terrequinone A (**26**).	[50]
*Aspergillus* *nidulans*	Deletion	Positive	Decreased production of sterigmatocystin (**16**) and norsolorinic aid (**27**).	[51]
*Aspergillus* *nidulans*	Deletion	Positive	Decreased production of streigmatocystin (**16**) and penicillin G (**28**).	[37]
*Aspergillus* *nidulans*	Deletion	Positive	Depressed expression of genes involved in biosynthesis of sterigmatocystin (**16**), terrequinone A (**26**) and penicillin G (**28**).	[52]
*Aspergillus* *nidulans*	Deletion	Negative	Increased production of sterigmatocystin (**16**).	[53]
*Aspergillus* *nidulans*	Overexpression	Positive	Increased production of sterigmatocystin (**16**).	[54]
*Aspergillus* *niger*	Deletion	Positive	Decreased production of asperrubrol (**29**), atromentin (**30**) and JBIR 86 (**31**).	[55]
*Aspergillus* *niger*	Deletion	Negative	Increased production of aspernigrin A (**32**) and BMS-192548 (**33**).	[55]
*Aspergillus* *niger*	Overexpression	Positive	Increased production of flaviolin (**34**), orlandin (**35**) and kotanin (**36**).	[56]
*Aspergillus* *niger*	Overexpression and deletion	Positive	Decreased production of OTA (**10**) in the deleted mutant, but increased production of OTA (**10**) in the overexpressed mutant.	[57]
*Aspergillus* *ochraceus*	Deletion	Positive	Decreased production of OTA (**10**).	[58]
*Aspergillus* *oryzae*	Deletion	Negative	Increased production of kojic acid (**20**).	[59]
*Aspergillus* *oryzae*	Overexpression	Positive	Increased production of monacolin K (**37**) and terrequinone A (**38**).	[60]
*Aspergillus* *pachycristatus*	Deletion	Positive	Decreased production of sterigmatocystin (**16**) and echinocandin B (**39**).	[61]
*Aspergillus* *pseudoterreus*	Overexpression	Positive	Increased production of itaconic acid (**40**).	[62]
*Aspergillus terreus*	Overexpression	Positive	Increased production of lovastatin (**41**).	[63]
*Aspergillus terreus*	Overexpression	Positive	Increased production of dihydroisoflavipucines 1 (**42**) and 2 (**43**).	[64]
*Beauveria* *bassiana*	Overexpression and deletion	Positive	Decreased production of beauvericin (**44**) and bassiatin (**45**) in the *BbLaeA* disruption strain, but increased production in the overexpressed strain.	[65]
*Botrytis* *cinerea*	Deletion	Positive	Decreased production of oxalic acid (**46**).	[66]
*Botrytis* *cinerea*	Deletion	Positive	Decreased production of abscisic acid (**47**).	[67]
*Chaetomium* *globosum*	Overexpression	Positive	Increased production of seven cytochalasans including chaetoglobosins A (**48**), B (**49**), D (**50**), E (**51**), O (**52**), V (**53**) and Z (**54**).	[68]
*Chaetomium* *globosum*	Overexpression and deletion	Positive	Decreased production of chaetoglobusin A (**48**) in Δ*CglaeA* mutant, restored production of chaetoglobusin A (**48**) in *CglaeA-C* strain, and increased production of chaetoglobusin A (**48**) in *CglaeA-OE* strain.	[69]
*Cladosporium* *fulvum*	Deletion	Negative	Increased production of cladofulvin (**55**).	[70]
*Cochliobolus* *heterostrophus*	Deletion	Positive	Decreased production of T-toxin (**56**).	[71]
*Coprinopsis* *cinerea*	Deletion	Negative	Increased production of coprinoferrin (**57**).	[72]
*Daldinia* *eschscholzii*	Replacement of a strong *pgdA* promoter	Positive	Induced production of dalestones A (**58**) and B (**59**).	[73]
*Dothistroma* *septosporum*	Deletion	Negative	Increased production of dothistromin (**60**).	[74]
*Fusarium* *fujikuroi*	Deletion	Negative	Increased production of bikaverin (**61**).	[75]
*Fusarium* *fujikuroi*	Deletion	Positive	Decreased production of gibberellins A3 (**63**) and A4 (**64**), fusarin C (**65**), fumonisins B1 (**66**), B2 (**67**), B3 (**68**) and B4 (**69**), deoxynivalenol (**70**), and 15-acetyl deoxynivalenol (**71**).	[75]
*Fusarium* *fujikuroi*	Deletion	Positive	Decreased production of fusaric acid (**62**), fusarinolic acid (**72**), and dehydrofusaric acid (**73**).	[76]
*Fusarium* *fujikuroi*	Deletion and overexpression	Positive	Deletion of *laeA* led to decreased production of gibberellins, fumonisins, and fusarin C (**65**). Overexpression of *laeA* led to increased production of gibberelins.	[77]
*Fusarium* *fujikuroi*	Deletion	Negative	Increased production of gibepyrones A (**74**), B (**75**), C (**76**), D (**77**), E (**78**), and F (**79**).	[78]
*Fusarium* *graminearum*	Deletion and overexpression	Positive	Deletion of *FglaeA* led to a dramatic reduced production of trichothecenes and zearalenone (**80**). Overexpression of *FglaeA* caused the increased production of trichothecenes and zearlenone (**80**).	[79]
*Fusarium* *oxysporum*	Deletion	Positive	Decreased production of beauvericin (**44**) and fusaric acid (**62**).	[80]
*Fusarium oxysporum* f.sp*. niveum*	Deletion	Positive	Decreased production of bikaverin (**61**) and fusaric acid (**62**).	[81]
*Fusarium* *verticillioides*	Deletion	Positive	Decreased production of bikaverin (**61**), fusaric acid (**62**), fusarin C (**65**), and fumonisins.	[82]
*Ganoderma* *lingzhi*	Deletion and overexpression	Positive	Decreased production of ganoderic acids in the deleted mutant and increased ganoderic acids in the overexpressed mutant.	[83]
*Magnaporthe* *oryzae*	Overexpression	Positive	Decreased production of melanin and increased production penicillin G (**28**).	[84]
*Magnaporthe* *oryzae*	Overexpression	Positive	Increased production of secondary metabolites.	[85]
*Monascus* *pilosus*	Overexpression	Positive	Increased production of monacolin K (**37**) and unidentified pigments.	[86]
*Monascus* *purpureus*	Overexpression	Positive	Increased production of monacolin K (**37**).	[87]
*Monascus* *ruber*	Deletion	Positive	Decreased production of citrinin (**81**) and six pigments rubropunctamine (**82**), monascorubramine (**83**), monascin (**84**), rubropunctatin (**85**), ankaflavin (**86**), and monascorubrin (**87**).	[88]
*Penicillium* sp. LC1-4	Overexpression	Positive	Increased production of quinolactacin A (**88**).	[29]
*Penicillium* sp. MB	Deletion	Positive	Inhibited production of the members with the 1-oxa-7-aza-spiro [4,4] non-2-ene-4,6-dione skeleton, including pseurotins A (**89**), B (**90**), C (**91**), D (**92**), and E (**93**).	[89]
*Penicillium brocae* HDN-12-143	Overexpression	Positive	Increased production of fumigatin chlorohydrin (**94**), iso-fumitatin chlorohydrin (**95**), spinulosin (**96**), and pyranonigrin F (**97**).	[90]
*Penicillium* *chrysogenum*	Overexpression and deletion	Positive	Overexpression of *PclaeA* gene led to increased production of penicillin G (**28**). Deletion of *PclaeA* led to decreased production of penicillin G (**28**).	[22]
*Penicillium* *chrysogenum*	Deletion	Positive	Decreased production of penicillin G (**28**).	[91]
*Penicillium* *chrysogenum*	Deletion	Positive	Decreased production of penicillin G (**28**).	[92]
*Penicillium* *chrysogenum*	Deletion	Positive	Small reduction in penicillin G (**28**).	[93]
*Penicillium* *citrinum*	Deletion	Positive	Decreased production of compactin (**98**).	[94]
*Penicillium* *digitatum*	Deletion	Positive	Reduced expression of several secondary metabolite BGCs.	[95]
*Penicillium* *dipodomyis* YJ-11	Overexpression	Positive	Increased production of sorbicillinoids including 10,11-dihydrobislongiquinolide (**99**), 10,11,16,17-tetrahydrobislongiquinolide (**100**), bislongiquinolide (**101**), 16,17-dihydrobislongiquinolide (**102**), sohirnone A (**103**), and 2′,3′-dihydrosorbicillin (**104**).	[96]
*Penicillium* *expansum*	Deletion	Positive	Decreased production of patulin (**105**).	[97,98]
*Penicillium* *oxalicum*	Deletion	Positive	Decreased production of secondary metabolites.	[99]
*Penicillium* *oxalicum*	Deletion	Positive	Four of the 28 secondary metabolite BGCs were significantly downregulated.	[100]
*Penicillium* *roqueforti*	Deletion	Positive	Decreased production of roquefortine C (**106**), mycophenolic acid (**107**), and andrastin A (**108**).	[101]
*Pestalotiopsis* *microspore*	Deletion	Positive	Decreased production of pestalotiollide B (**109**).	[102]
*Pleurotus* *ostreatus*	Deletion	Positive	Decreased production of the intracellular polysaccharide (IPS).	[103]
*Pyricularia* *oryzae*	Deletion and overexpression	Positive	Deletion of *PoLAE1* reduced the production of tenuazonic acid (**110**). Overexpression of *PoLAE1* led to increased production of tenuazonic acid (**110**).	[104]
*Trichoderma* *afroharzianum*	Overexpression	Positive	Induced production of (1*R*,*3E*,*5E*)-1-(3,5-dihydroxy- 2,4-dimethylphenyl)-1-hydroxyhepta- 3,5-dien-2-one (**111**) and (1*R*,*3E*,*5E*)-1-(3,5-dihydroxy- 2,4-dimethylphenyl)-1-methoxyhepta- 3,5-dien-2-one (**112**).	[105]
*Trichoderma* *longibrachiatum*	Deletion and overexpression	Positive	Deletion of *Tllae1* reduced the production of peptaibols. Overexpression of *Tllae1* led to two-fold increased production of petaibols.	[106]
*Trichoderma* *reesei*	Overexpression	Positive	Increased production of sorbicillinoids.	[107]
*Trichoderma* *reesei*	Deletion	Positive	Decreased production of sterigmatocystin (**16**).	[108]
*Trichoderma* *reesei*	Deletion	Negative	Increased production of ophiobolin F (**113**).	[109]
*Valsa mali*	Deletion	Positive	Decreased production of toxic metabolites.	[110]

**Table 2:** Some examples of VeA regulating secondary metabolite production in fungi.

Fungus	Overexpression/ Deletion of *veA*	Positive/Negative Regulation	Production of Secondary Metabolites	Ref.
*Acremonium* *chrysogenum*	Deletion	Positive	Decreased production of cephalosporin C (**114**).	[133]
*Alternaria* *alternata*	Deletion	Positive	Decreased production of alternariol (**4**) and alternariol monomethyl ether (**5**).	[26]
*Alternaria* *alternata*	Deletion	Positive	Decreased production of alternariol (**4**) and alternariol monomethyl ether (**5**).	[134]
*Aspergillus* *carbonarius*	Deletion	Positive	Production of ochratoxin A (**10**) was decreased to almost zero.	[32]
*Aspergillus* *carbonarius*	Deletion	Positive	Decreased production of ochratoxin A (**10**).	[135]
*Aspergillus* *flavus*	Deletion	Positive	Decreased production of cyclopiazonic acid (**18**), aflatrem B (**115**), and aflatoxins.	[136]
*Aspergillus* *flavus*	Deletion	Positive	Decreased production of asparasone A (**116**).	[137]
*Aspergillus* *flavus*	Deletion	Positive	Decreased production of aflatoxin B1 (**17**).	[138]
*Aspergillus* *flavus*	Deletion and overexpression	Positive	Decreased production of aflatoxins in the deletion mutant and increased production of aflatoxins in the overexpression mutant.	[139]
*Aspergillus* *fumigatus*	Deletion	Positive	Decreased production of gliotoxin (**21**).	[140]
*Aspergillus* *fumigatus*	Overexpression	Negative	Decreased production of gliotoxin (**21**).	[140]
*Aspergillus* *fumigatus*	Deletion	Positive	Decreased production of fumagillin (**23**), gumitremorgin G (**117**), fumigaclavine C (**118**), and glionitrin A (**119**).	[141]
*Aspergillus* *fumigatus*	Overexpression	Negative	Decreased production of fumagillin (**23**), gumitremorgin G (**117**), fumigaclavine C (**118**), and glionitrin A (**119**).	[141]
*Aspergillus* *nidulans*	Deletion	Positive	Decreased production of sterigmatocystin (**16**) and penicillin G (**28**).	[142]
*Aspergillus* *nidulans*	Deletion	Positive	Decreased production of penicillin G (**28**).	[143]
*Aspergillus* *nidulans*	Deletion	Positive	Decreased production of sterigmatocystin (**16**) and norsolorinic aid (**27**)	[51]
*Aspergillus* *nidulans*	Deletion	Negative	Increased production of orsellinic acid (**120**), F9775A (**121**) and F9775B (**122**).	[144]
*Aspergillus* *nidulans*	Deletion	Positive	Decreased production of aspernidines.	[145]
*Aspergillus* *niger*	Deletion	Positive	Decreased production of ochratoxins A (**10**), α (**123**), and β (**124**).	[146]
*Aspergillus* *ochraceus*	Deletion	Positive	Decreased production of ochratoxin A (**10**).	[58]
*Aspergillus* *oryzae*	Deletion	Positive	Decreased production of kojic acid (**20**).	[147]
*Aspergillus* *pachycristatus*	Deletion	Positive	Decreased production of sterigmatocystin (**16**) and echinocandin B (**39**).	[61]
*Aspergillus* *parasiticus*	Deletion	Positive	Decreased production of sterigmatocystin (**16**).	[148]
*Aspergillus* *parasiticus*	Deletion	Positive	Reduced aflatoxin BGC gene expression and aflatoxisome development.	[149]
*Chaetomium* *globosum*	Deletion and overexpression	Positive	Decreased production of chaetoglobusin A (**48**) in the *veA* deleted mutant and increased production of chaetoglobusin A (**48**) in the *veA* overexpressed mutant.	[150]
*Cladosporium* *fulvum*	Deletion	Negative	Increased production of cladofulvin (**55**).	[70]
*Cochliobolus* *heterostrophus*	Deletion	Positive	Decreased production of T-toxin (**56**).	[71]
*Fusarium* *fujikuroi*	Deletion	Negative	Increased production of bikaverin (**61**).	[75]
*Fusarium* *fujikuroi*	Deletion	Positive	Decreased production of gibberellins A3 (**63**) and A4 (**64**), fusarin C (**65**), fumonisins B1 (**66**), B2 (**67**), B3 (**68**) and B4 (**69**), deoxynivalenol (**70**), and 15-acetyl deoxynivalenol (**71**).	[75]
*Fusarium* *fujikuroi*	Deletion	Positive	Decreased production of fusaric acid (**62**), fusarinolic acid (**72**), and dehydrofusaric acid (**73**)	[76]
*Fusarium* *fujikuroi*	Deletion	Positive	Decreased production of gibberellins, fumonisins, and fusarin C (**65**).	[77]
*Fusarium* *fujikuroi*	Deletion	Negative	Increased production of gibepyrones A (**74**), B (**75**), C (**76**), D (**77**), E (**78**), and F (**79**).	[78]
*Fusarium* *graminearium*	Deletion	Positive	Reduced production of deoxynivalenol (**69**).	[151]
*Fusarium* *graminearium*	Deletion	Positive	Decreased production of trichothecenes.	[152]
*Fusarium* *nematophilum*	Overexpression	Positive	Increased production of antitumor compounds.	[153]
*Fusarium* *oxysporum*	Deletion	Positive	Decreased production of beauvericin (**44**) and fusaric aicd (**73**)	[80]
*Fusarium* *oxysporum* f.sp. *niveum*	Deletion	Positive	Decreased production of bikaverin (**61**) and fusaric acid (**62**)	[81]
*Fusarium* *verticillioides*	Deletion	Positive	Decreased production of fusarin C (**65**) and fumonisins B1 (**66**), B2 (**67**) and B3 (**68**).	[154]
*Mycosphaerella* *graminicola*	Deletion	Positive	Decreased production of melanin.	[155]
*Neurospora* *crassa*	Deletion	Positive	Decreased production of carotenoids.	[156]
*Neurospora* *crassa*	Deletion	Positive	Decreased production of siderophore coprogen (**125**) and carotenoids.	[157]
*Penicillium* *chrysogenum*	Deletion	Positive	Decreased production of penicillin G (**28**).	[91]
*Penicillium* *chrysogenum*	Deletion	Positive	Decreased production of penicillin G (**28**).	[158]
*Penicillium* *chrysogenum*	Deletion	Positive	Small reduction in penicillin G (**28**).	[93]
*Penicillium* *citrinum*	Deletion and overexpression	Positive	Decreased production of compactin (**98**) in the deletion mutant, and increased production of compactin (**98**) in the overexpressed mutant.	[94]
*Penicillium* *citrinum*	Overexpression	Positive	Increased production of compactin (**98**).	[159]
*Penicillium* *expansum*	Deletion	Positive	Decreased production of citrinin (**81**) and patulin (**105**).	[127]
*Penicillium* *expansum*	Deletion	Positive	Blocked production of patulin (**105**).	[160]
*Penicillium* *expansum*	Deletion	Positive	Lost production of patulin (**105**).	[161]
*Pestalotiopsis* *microspora*	Deletion	Negative	Increased production of pestalotiollide B (**103**).	[102]

**Table 3:** Some examples of VelB regulating secondary metabolite production in fungi.

Fungus	Overexpression/ Deletion of *velB*	Positive/Negative Regulation	Production of Secondary Metabolites	Ref.
*Aspergillus* *flavus*	Deletion	Positive	Decreased production of aflatoxins.	[167]
*Aspergillus* *flavus*	Deletion	Positive	Decreased production of aflatoxin B1 (**17**).	[138]
*Aspergillus* *nidulans*	Deletion	Positive	Decreased production of sterigmatocystin (**16**).	[128]
*Aspergillus* *nidulans*	Deletion	Negative	Increased production of sterigmatocystin (**16**).	[168]
*Aspergillus ochraceus*	Deletion	Positive	Reduced production of ochratoxin A (**10**).	[58]
*Aspergillus* *oryzae*	Deletion	Negative	Increased production of kojic acid (**20**).	[147]
*Botrytis* *cinerea*	Deletion	Negative	Increased production of melanin.	[169]
*Colletotrichum* *siamense*	Deletion	Positive	Decreased production of melanin.	[170]
*Curvularia* *lunata*	Deletion	Positive	Decreased production of methyl 5-hydroxymethylfuran-2-carboxylate (**126**).	[171]
*Fusarium* *fujikuroi*	Deletion	Positive	Decreased production of gibberellins, fumonisins and fusarin C (**65**).	[77]
*Fusarium* *fujikuroi*	Deletion	Negative	Increased production of gibepyrones A (**74**), B (**75**), C (**76**), D (**77**), E (**78**), and F (**79**)	[78]
*Fusarium* *graminearum*	Deletion	Positive	Decreased production of deoxynivalenol (**68**).	[171]
*Fusarium* *graminearum*	Deletion	Positive	Decreased production of trichothecenes and zearalenone (**80**).	[172]
*Fusarium* *pseudograminearum*	Deletion	Positive	Decreased production of deoxynivalenol (**68**).	[173]
*Neurospora* *crassa*	Deletion	Positive	Reduced biosynthesis of carotenoids.	[157]
*Penicillium* *chrysogenum*	Deletion	Negative	Increased production of penicillin G (**28**).	[174]
*Penicillium* *Expansum*	Deletion	Positive	Blocked production of patulin (**105**).	[160]
*Penicillium* *expansum*	Deletion	Positive	Decreased production of chaetoglobusin A (**48**), citrinin (**81**), and patulin (**105**).	[175]
*Penicillium* *expansum*	Deletion	Negative	Increased production of fumarylalanine (**127**).	[175]
*Pestalotiopsis* *microspora*	Deletion	Positive	Decreased production of pestalotiollide B (**103**).	[102]

**Table 4:** Some examples of VelC, VelD, and Vos A regulating secondary metabolite production in fungi.

Overexpression/ Deletion	Fungus	Positive/Negative Regulation	Production of Secondary Metabolites	Ref.
Deletion of *velC*	*Aspergillus* *Oyzae*	Positive	Decreased production of kojic acid (**20**).	[147]
Deletion of *velC*	*Penicillium* *Expansum*	Positive	Decreased production of patulin (**105**).	[160]
Deletion of *velD*	*Aspergillus* *flavus*	Positive	Decreased production of aflatoxin B1 (**17**).	[138]
Deletion of *velD*	*Aspergillus* *oryzae*	Positive	Decreased production of kojic acid (**20**).	[147]
Deletion of *vosA*	*Aspergillus* *nidulans*	Positive	Lost production of trehalose (**128**) in spores.	[177]
Deletion of *vosA*	*Aspergillus* *nidulans*	Negative	Slightly increased production of sterigmatocystin (**16**) in ascospores and upregulation of the BGCs associated with the biosynthesis of other secondary metabolites, including asperthecin (**129**), microperfuranone (**130**), and monodictyphenone (**131**).	[178]
Deletion of *vosA*	*Aspergillus* *nidulans*	Negative	Increased production of emericellamides A (**132**), C (**133**) and D (**134**), austinol (**135**) and dehydroaustinol (**136**).	[125]
Deletion of *vosA*	*Aspergillus* *nidulans*	Negative	Increased production of sterigmatocystin (**16**).	[179]
Deletion of *vosA*	*Aspergillus* *oryzae*	Negative	Increased production of kojic acid (**20**).	[147]

## Supplementary Materials

The following supporting information can be downloaded at: https://www.mdpi.com/article/10.3390/jof10080561/s1, Figure S1: Structures of the compounds **1**–**141** identified from fungi through regulation of LaeA and velvet proteins.
