# Supplementary material for: Involvement of LaeA and Velvet Proteins in Regulating the Production of Mycotoxins and Other Fungal Secondary Metabolites
Source: J Fungi (Basel). 2024 Aug 8;10(8):561. doi: 10.3390/jof10080561 (PMC11355368; doi:10.3390/jof10080561)
Supplement: Supplementary file 1 [file jof-10-00561-s001.zip › jof-3129517-supplementary.pdf]

Supplementary Materials:

Involvement of LaeA and Velvet Proteins in Regulating the Production of Mycotoxins and Other Fungal Secondary Metabolites

Xuwen Hou, Liyao Liu, Dan Xu, Daowan Lai, and Ligang Zhou *

Department of Plant Pathology and MOA Key Lab of Pest Monitoring and Green Management, College of Plant Protection, China Agricultural University, Beijing 100193, China; xwhou@cau.edu.cn (X.H); lyliu@cau.edu.cn (L.L); cauxudan@cau.edu.cn (D.X.); dwlai@cau.edu.cn (D.L.)

* Correspondence: lgzhou@cau.edu.cn (L.Z.); Tel.: +86-10-6273-1199

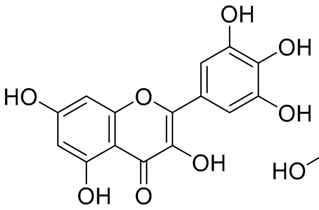

1. Myricetin

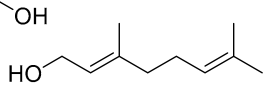

2. Geraniol

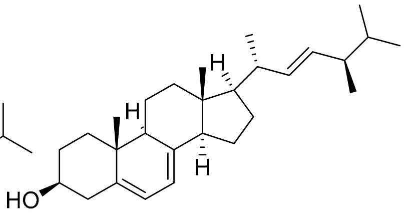

3. Ergosterol

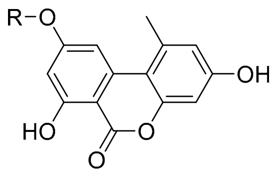

4. Alternariol, R=H
5. Alternariol monomethyl ether, R=CH_3

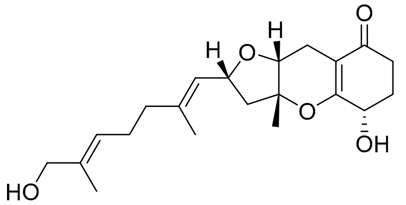

6. Tricycloalternarene O

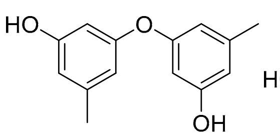

7. Diorcinol

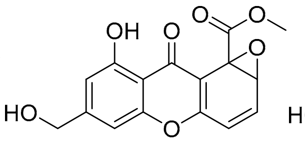

8. MS-347a

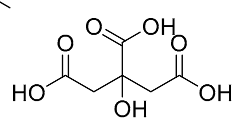

9. Citric acid

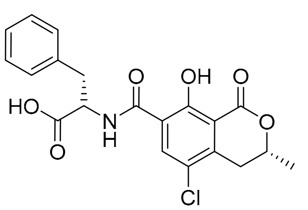

10. Ochratoxin A

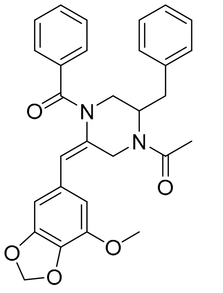

11. Flavipanmide A

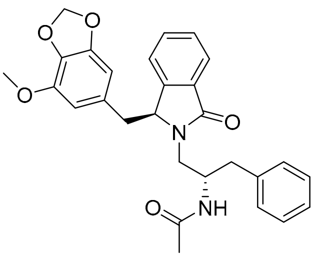

12. Flavipanmide B

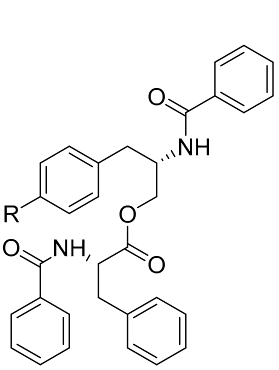

13. Asperphenamate, R=H
14. 4'-OMe-Asperphenamate, R=OCH_3

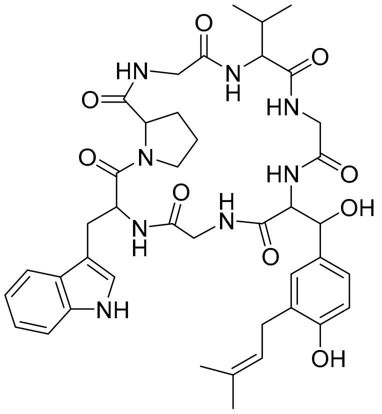

15. Cyclic Pro-Gly-Val-Gly-Try (8-OH, 3-prenyl)-Gly-Trp

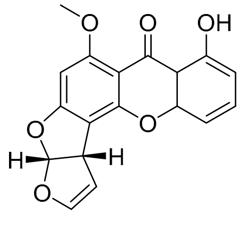

16. Sterigmatocystin

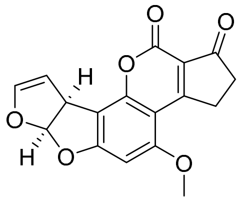

17. Aflatoxin B1

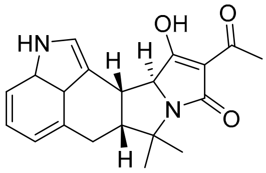

18. Cyclopiazonic acid

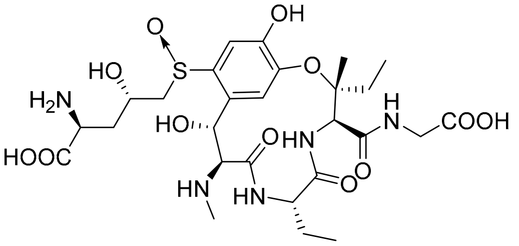

19. Ustiloxin B

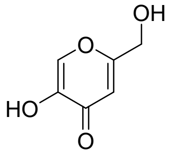

20. Kojic acid

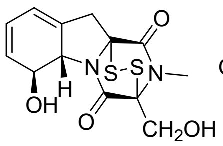

21. Gliotoxin

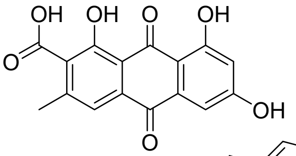

22. Endocrocin

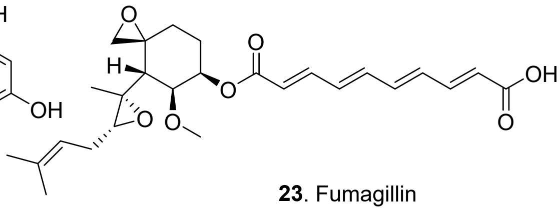

23. Fumagillin

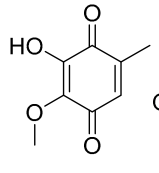

24. Fumigatin

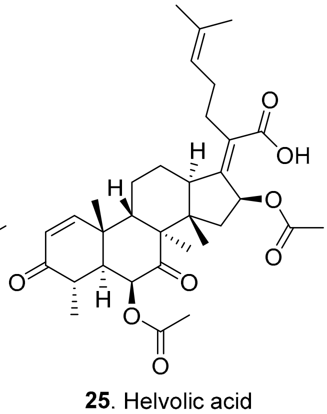

25. Helvolic acid

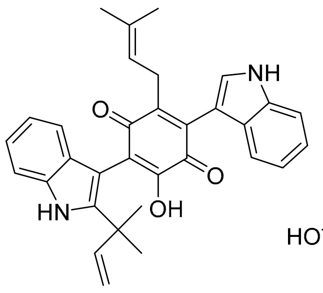

26. Terrequinone A

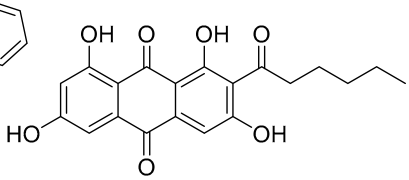

27. Norsolorinic acid

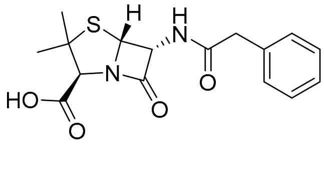

28. Penicillin G

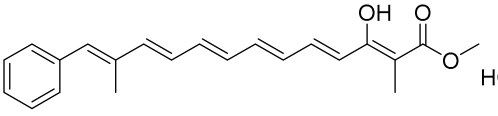

29. Asperrubrol

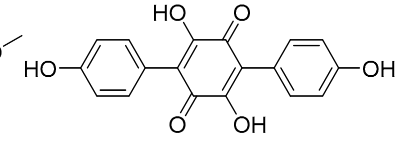

30. Atromentin

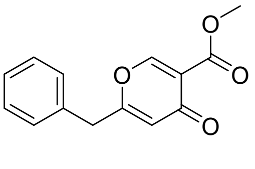

31. JBIR 86

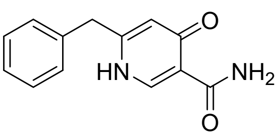

32. Aspernigrin A

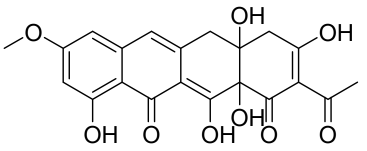

33. BMS-192548

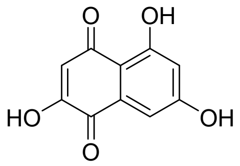

34. Flaviolin

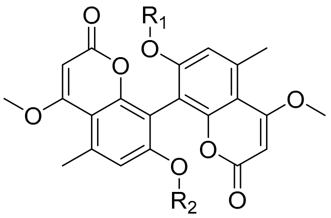

35. Orlandin, R_1=H, R_2=H
36. Kotanin, R_1=CH_3, R_2=CH_3

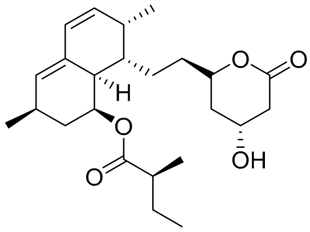

37. Monacolin K

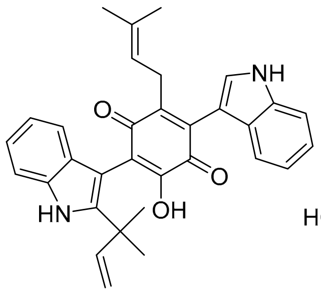

38. Terrequinone A

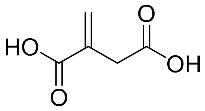

40. Itaconic acid

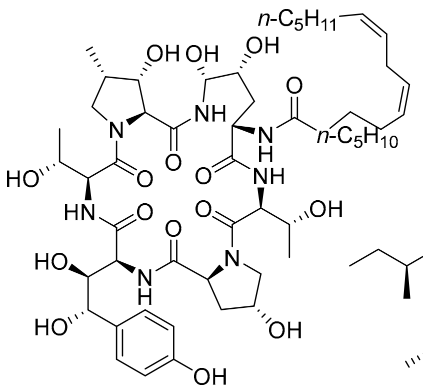

39. Echinocandin B

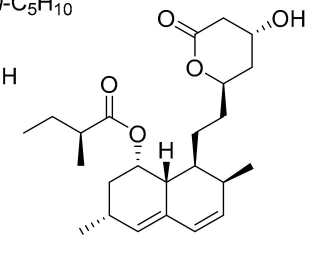

41. Lovastatin

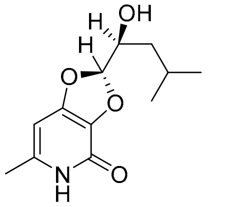

42. Dihydroisoflavipucine 1

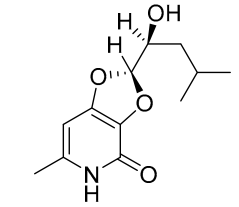

43. Dihydroisoflavipucine 2

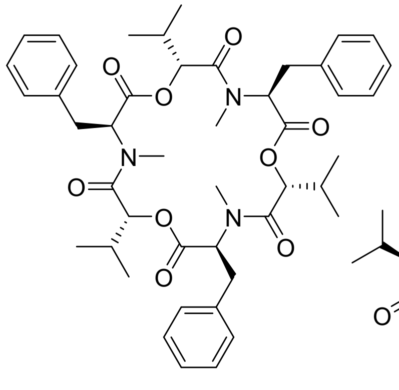

44. Beauvericin

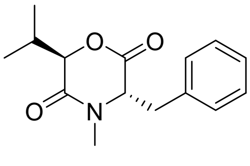

45. Bassiatin

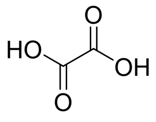

46. Oxalic acid

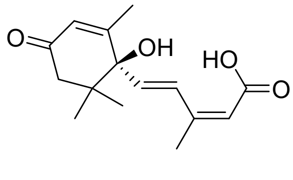

47. Abscisic acid

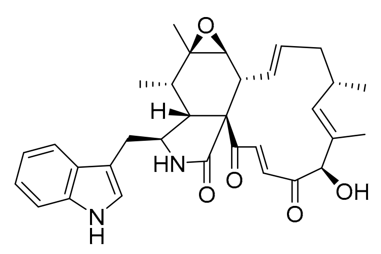

48. Chaetoglobosin A

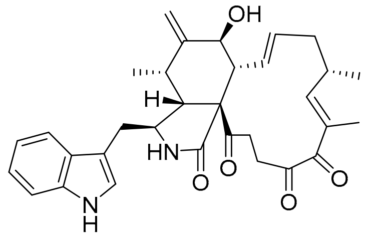

49. Chaetoglobosin B

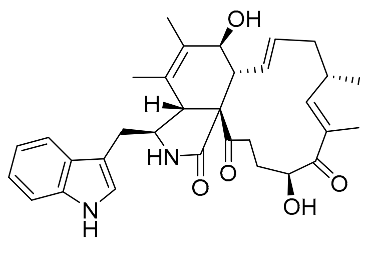

50. Chaetoglobosin D

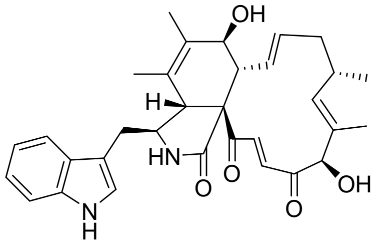

51. Chaetoglobosin E

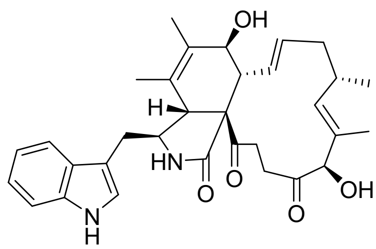

52. Chaetoglobosin O

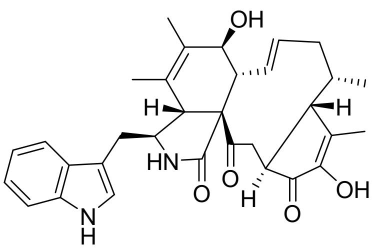

53. Chaetoglobosin V

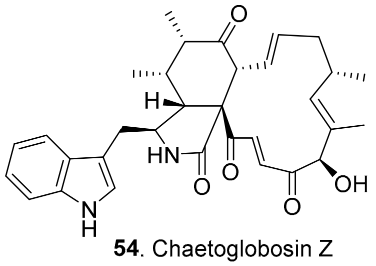

54. Chaetoglobosin Z

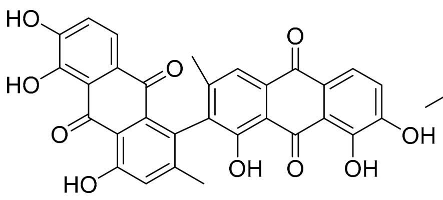

55. Cladofulvin

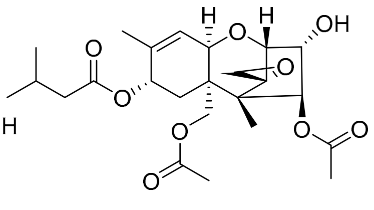

56. T-toxin

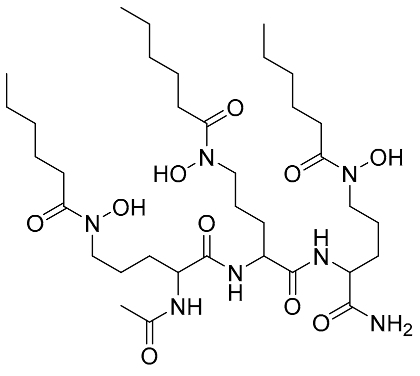

57. Coprinoferrin

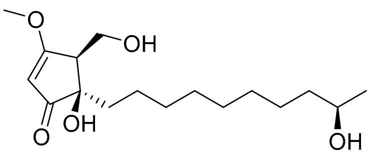

58. Dalestone A

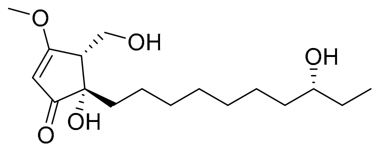

59. Dalestone B

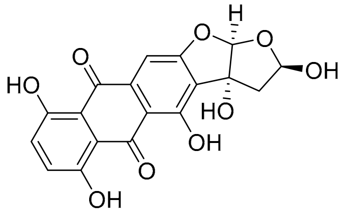

60. Dothistromin

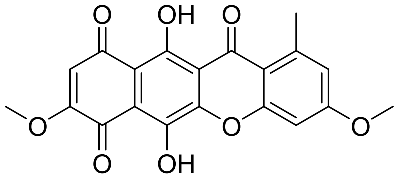

61. Bikaverin

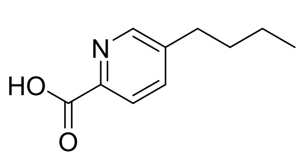

62. Fusaric acid

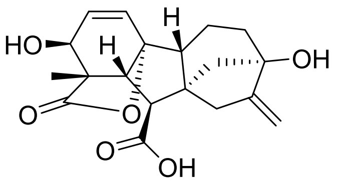

63. Gibberellin A3

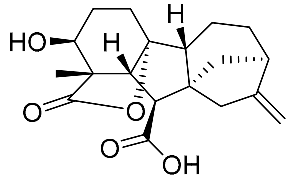

64. Gibberellin A4

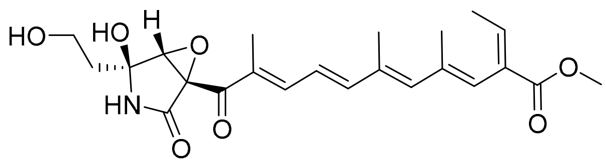

65. Fusarin C

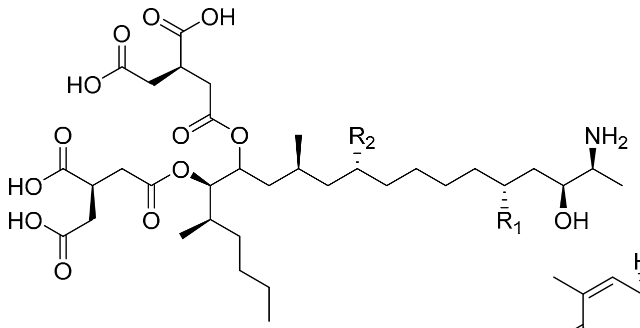

66. Fumonisin B1, R_1=OH, R_2=OH
67. Fumonisin B2, R_1=OH, R_2=H
68. Fumonisin B3, R_1=H, R_2=OH
69. Fumonisin B4, R_1=H, R_2=H

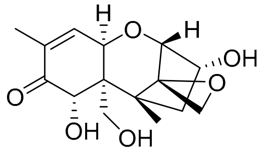

70. Deoxynivalenol

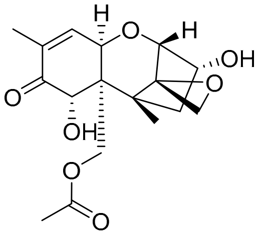

71. 15-Acetyl deoxynivalenol

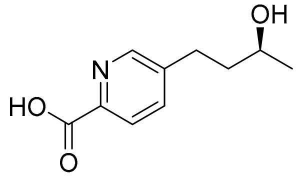

72. Fusarinolic acid

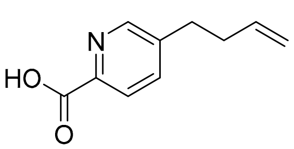

73. Dehydrofusaric acid

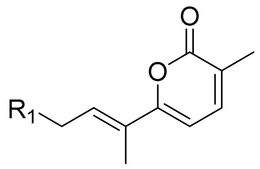

74. Gibepyrone A. R=H
75. Gibepyrone B, R=OH

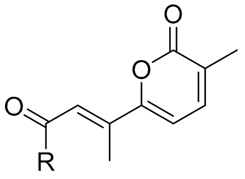

76. Gibepyrone C, R=H
77. Gibepyrone D, R=OH

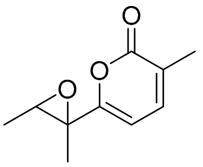

78. Gibepyrone E

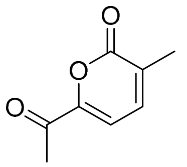

79. Gibepyrone F

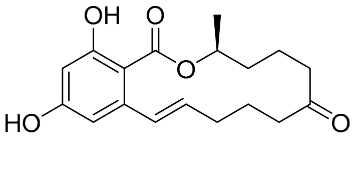

80. Zearalenone

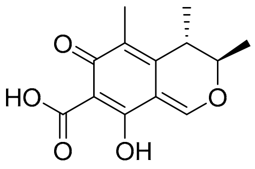

81. Citrinin

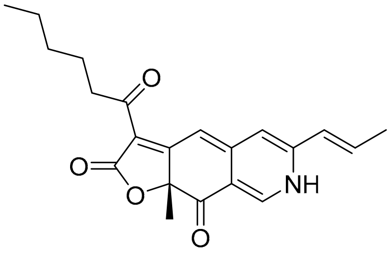

82. Rubropunctamine

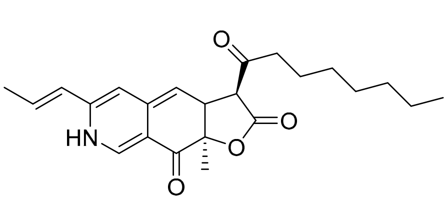

83. Monascorubramine

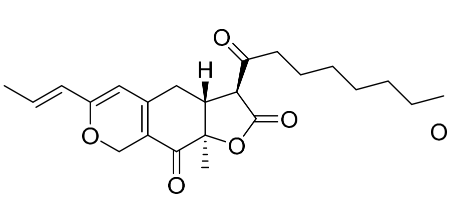

84. Monascin

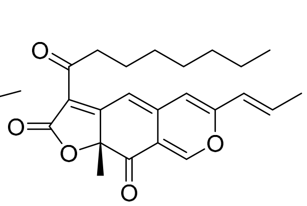

85. Rubropunctatin

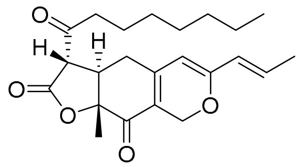

86. Ankaflavin

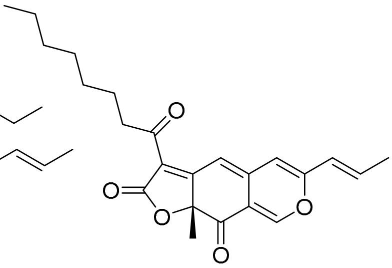

87. Monascorubin

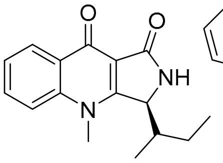

88 Quinolactacin A

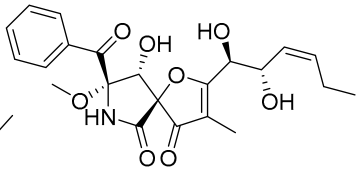

89. Pseurotin A

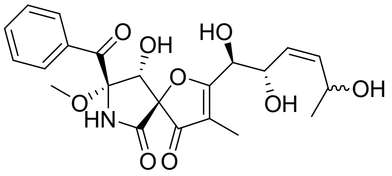

90. Pseurotin B

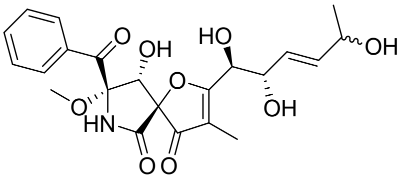

91. Pseurotin C

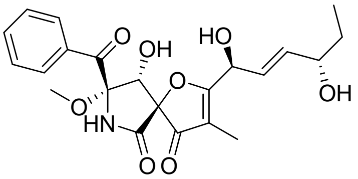

92. Pseurotin D

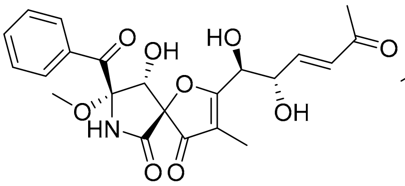

93. Pseurotin E

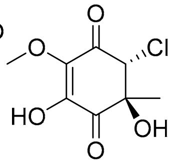

94. Fumigatin chlorohydrin

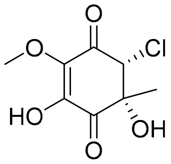

95. Iso-fumigatin chlorohydrin

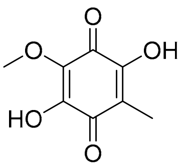

96. Spinulosin

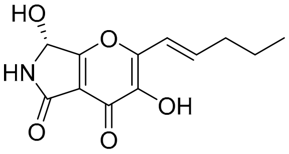

97. Pyranonigrin F

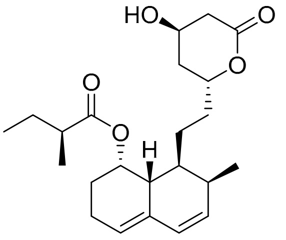

98. Compactin

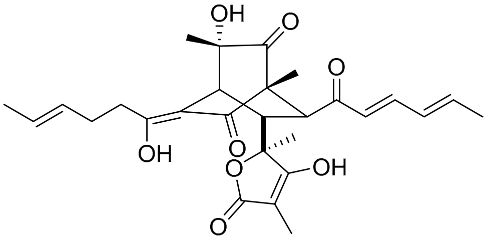

99. 10,11-Dihydrobislongiquinolide

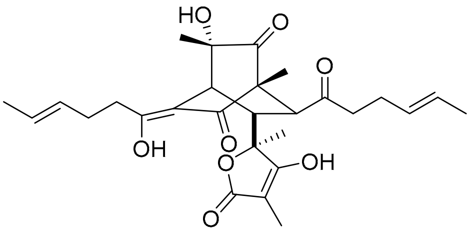

100. 10,11,16,17-Tetrahydrobislongiquinolide

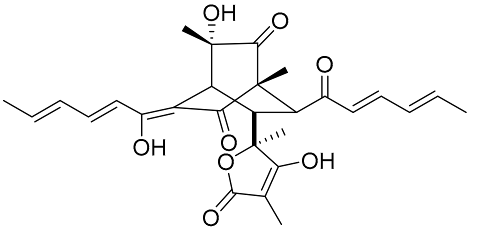

101. Bisorbibutenolide

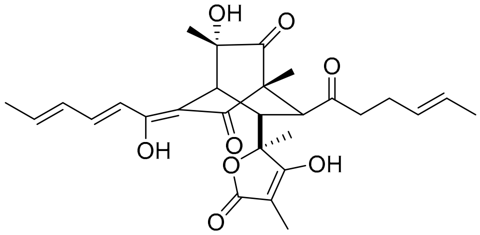

102. Dihydrotrichotetronine (16,17-dihydrobislongiquinolide)

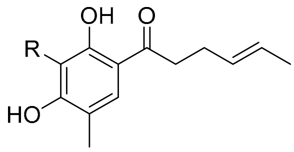

103. Sohirnone A, R=H
104. 2',3'-Dihydrosorbicillin, R=CH_3

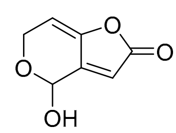

105. Patulin

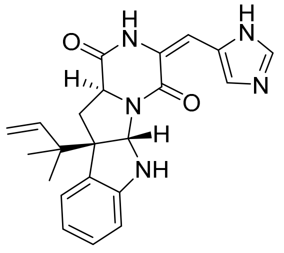

106. Roquefortine C

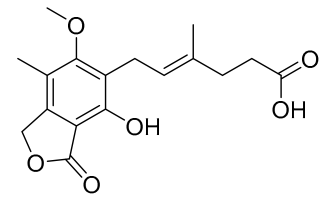

107. Mycophenolic acid

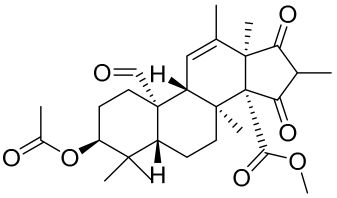

108. Andrastin A

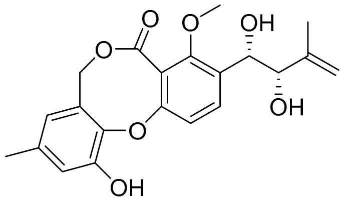

109. Pestalotiollide B

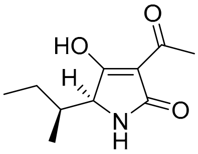

110. Tenuazonic acid

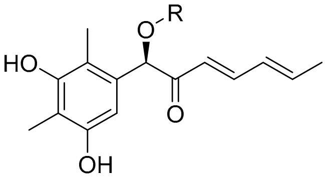

111. (1*R*,3*E*,5*E*)-1-(3,5-Dihydroxy-2,4-dimethyl phenyl)-1-hydroxyhepta-3,5-dien-2-one, R=H

112. (1*R*,3*E*,5*E*)-1-(3,5-Dihydroxy-2,4-dimethyl phenyl)-1-hydroxyhepta-3,5-dien-2-one, R=OH

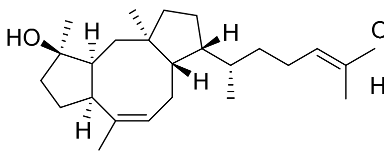

113. Ophiobolin F

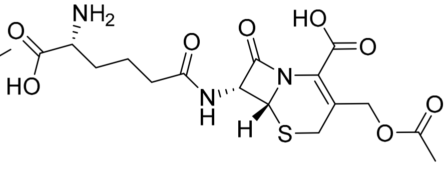

114. Cephalosporin C

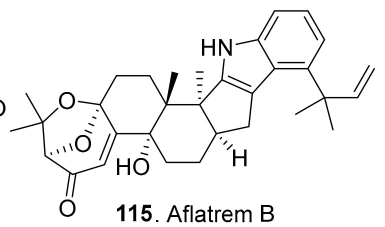

115. Aflatrem B

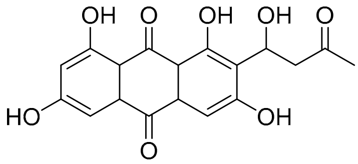

116. Asparasone A

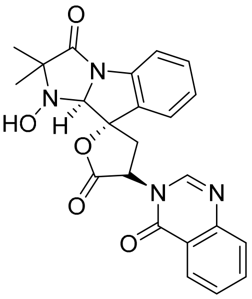

117. Gumitremorgin G

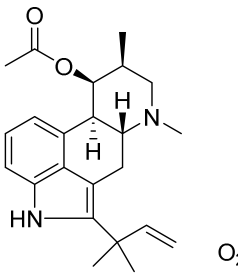

118. Fumigaclavine C

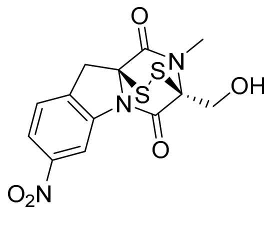

119. Glionitrin A

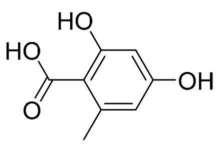

120. Orsellinic acid

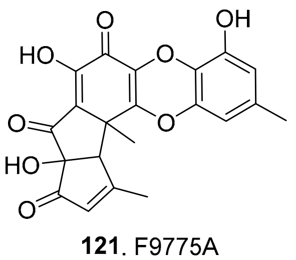

121. F9775A

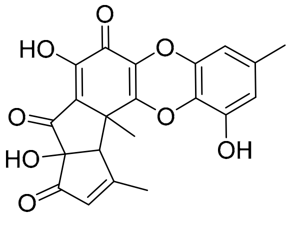

122. F9775B

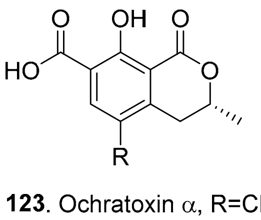

123. Ochratoxin α, R=Cl

124. Ochratoxin β, R=H

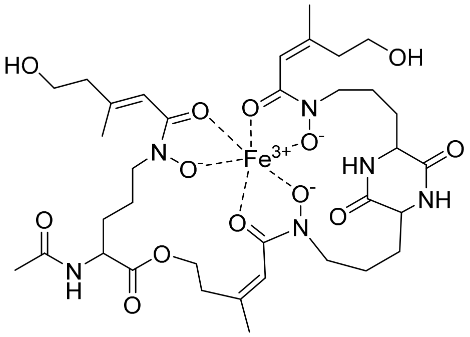

125. Siderophore coprogen

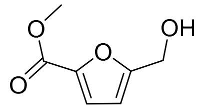

126. Methyl 5-hydroxymethyl furan-2-carboxylate

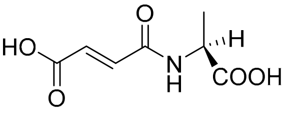

127. Fumarylalanine

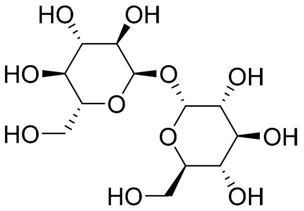

128. Trehalose

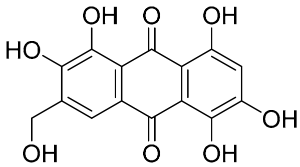

129. Asperthecin

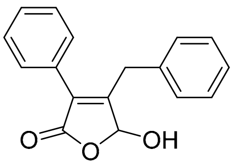

130. Microperfuranone

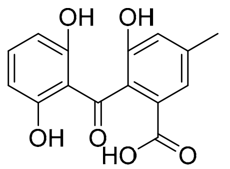

131. Monodictyphenone

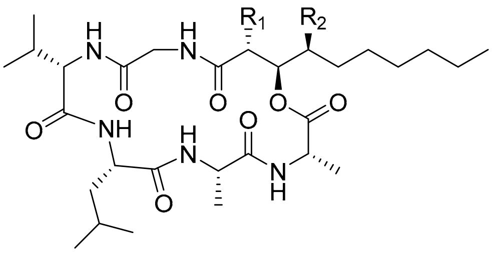

132. Emericellamide A, R_1=R_2-CH_3
133. Emericellamide C, R_1=CH_3, R_2=H
134. Emericellamide D, R_1=H, R_2=CH_3

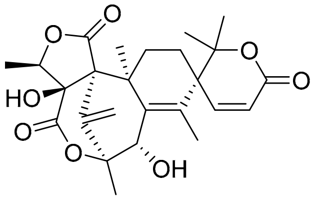

135. Austinol

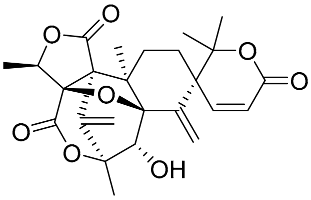

136. Dehydroaustinol

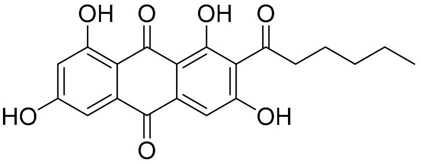

137. Norsolorinic acid

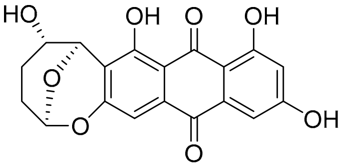

138. Nidurufin

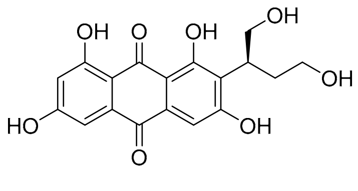

139. Versiconol

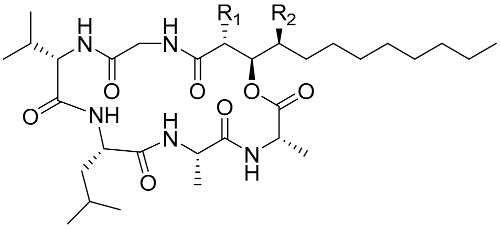

140. Emericellamide E, R_1=CH_3, R_2=H
141. Emericellamide F, R_1=H, R_2=CH_3

Figure S1. Structures of the metabolites **1–141** identified from fungi through regulation of LaeA and velvet proteins.
